# Directions of Changes in the Profession of Hospital Pharmacist in Poland

**DOI:** 10.3390/ijerph192114522

**Published:** 2022-11-05

**Authors:** Marcin Bochniarz, Elżbieta Inglot-Brzęk, Anna Lewandowska, Joanna Podgórska

**Affiliations:** 1 Specialist Hospital, Subcarpathian Oncology Centre, 36-200 Brzozów, Poland; 2 OnData Spółka z o.o., 35-030 Rzeszów, Poland; 3 Department of Management, University of Information Technology and Management, 35-225 Rzeszów, Poland; 4 Department of Economics and Finance, University of Information Technology and Management, 35-225 Rzeszów, Poland

**Keywords:** management, healthcare organizations, hospital pharmacist, innovation

## Abstract

The Act on the Pharmacist Profession, adopted on 10 December 2020, is a breakthrough for the entire community of pharmacists in Poland. Due to the scope of the changes introduced in the Act, the question is whether pharmacists in Poland are suitably prepared for pharmaceutical care and clinical pharmacy services. The main aim of the study is to assess the readiness of hospital pharmacists to introduce changes in the way the profession functions. The result of the study is the presentation of the concept of a new model of pharmacist functioning in healthcare entities and the indication of actions necessary to carry out such a change. The questionnaire was addressed to all hospital pharmacists in Poland. Two hundred and seventy-seven hospital pharmacists were included in the research. The analysis of the data revealed that almost all (96.1%) respondents indicated the need to introduce changes to the model of functioning of pharmacists in medical entities. Two-thirds of pharmacists declared readiness to start work to introduce changes. Hospital pharmacists recognize the importance of clinical pharmacy in their current practice; however, the biggest barrier is the lack of financing, an insufficient number of staff, a lack of knowledge and skills, and a lack of tools to use the service.

## 1. Introduction

The overriding goal of the hospital pharmacist profession is to optimize patient outcomes through joint, interdisciplinary activities aimed at the responsible use of drugs and medical devices [1]. Historically, the pharmacy’s tasks were focused on the production and provision of access to high-quality medicinal products. However, over the years, pharmacy has undergone many changes, and thus, the profession of pharmacist has also changed [2,3,4]. During this time, Polish pharmacists began to deal mainly with distribution and accounting tasks. Thus, this profession in Poland was limited to drug-selling activities [5,6]. Nowadays, pharmacists practice in a variety of settings—community and hospital pharmacies, regulatory and health authorities, the pharmaceutical industry, and research—and are generally recognized as drug experts regardless of their specific role.

In response to new challenges, clinical pharmacy has developed in the world as a tool aimed at the pharmacotherapy of an individual patient. The recipient of clinical pharmacy solutions is not the population, but the individual patient. The subject, in turn, is not the medicinal product, but the necessity, combination, and manner of administering the medicinal products to that patient. The concept of hospital pharmacy described in the Basel Declaration [1] distinguishes the following areas: procurement; influences on prescribing; preparation and delivery; administration; monitoring of medicines’ uses; human resources, training, and development.

The Act on the Pharmacist Profession [7], adopted on 10 December 2020, is a breakthrough for the entire community of pharmacists and a great challenge. Among other things, this Act enables pharmacists to provide pharmaceutical care and clinical pharmacy services to patients. The range of pharmaceutical services gradually developed and implemented in pharmacies enables a holistic approach to disease, the rationalization of pharmacotherapy, and the improvement of patients’ health comfort, as well as the rationalization of expenses for healthcare. Similar solutions are successfully used in many places, including in Italy, Denmark, Belgium, Great Britain [8], or Canada, where, as part of medication reviews dedicated to patients with chronic diseases, the pharmacist contributes to a significant improvement in medical recommendations and the cost-effectiveness of pharmacotherapy [9]. Due to the scope of the changes introduced in the Act, the question is whether pharmacists in Poland are properly prepared for pharmaceutical care and clinical pharmacy services [10].

Pharmacists must cope with the demands of different roles as well as increasing pressures such as managing healthcare and primary care. Tracking and responding to the wellbeing of pharmacists related to work is necessary because of its impact on the standard of pharmacy services [11,12,13]. The work-related wellbeing of pharmacists can be tested for negative (e.g., stress) and positive (e.g., eustress) outcomes; see [14].

In 2020, the Ministry of Health produced the report “Pharmaceutical care. Comprehensive analysis of the implementation process” [15], the aim of which was to present models and solutions for pharmaceutical care that can be successfully implemented in Poland. However, there are no studies that would empirically verify the degree of preparedness of hospital pharmacists to apply the changes resulting from the adopted Act.

The aim of this study is to propose a model for changing the scope of the tasks, role, and functioning of hospital pharmacists (HPhs) in healthcare entities after the entry into force of the Act of 10 December 2020 on the Pharmacist Profession [7]. This Act sanctions many new activities of pharmacists, including activities in the clinical area, i.e., clinical pharmacists’ services, the recipient of which is an individual patient.

The main aim of the study is to assess the readiness of hospital pharmacists to introduce changes in the way the profession functions. The result of the study is therefore the presentation of the concept of a new model of pharmacist functioning in healthcare entities and an indication of actions necessary to introduce changes in this area. There are four key questions regarding this goal:

- What is the satisfaction rating with the way a pharmacist currently performs his profession in a medical entity?
- What is the assessment of the need to introduce changes in the manner of performing the profession of pharmacist in medical entities?
- What are the areas of change in hospital pharmacy and clinical pharmacy?
- What are the problems related to the introduction of clinical pharmacy?

The study is based on data collected in a survey among 277 hospital pharmacists in Poland. The survey was conducted between January and February 2022 using the Surveymonkey website.

The remainder of the article is organized as follows: the next section provides a literature review on the current status of hospital pharmacy; then, the next section describes the hospital pharmacy solutions. In the fourth section, the authors present the description of methods and the data used. The fifth section consists of the presentation and discussion of the results. The paper ends with the concluding remarks.

## 2. Status of Hospital Pharmacy

The legal Act regulating the functioning of hospital pharmacies in Poland is the Pharmaceutical Law. It specifies the requirements for pharmacies and hospital pharmacy departments, the scope of services provided, and the tasks of the manager. The second legal Act regulating the area of hospital pharmacy activity is the Act on the profession of pharmacist, which specifies, inter alia, the objectives and scope of practicing the profession of a pharmacist, including working in a hospital or company pharmacy.

At present, in relation to hospital and company pharmacies as well as hospital pharmacy departments, there is a minimum employment standard, which establishes the minimum human resources necessary for the operation of these organizational units.

As of 1 September 2022, there were 565 hospital and company pharmacies and 1038 hospital pharmacy departments operating in Poland [16]. The latest data from Centrum e-Zdrowie on the employment of hospital pharmacists (as of 31 December 2020) indicate that 2090 pharmacists are employed in medical entities in Poland. At the same time, according to the data from the Register of Healthcare Entities (in Polish: Rejestr Podmiotów Wykonujących Działalność Leczniczą, RPWDL), as of 16 August 2022, there were 225,628 hospital beds in Poland.

The analysis of the above data shows that in the third quarter of 2022, approximately 730 pharmacist positions in hospitals are lacking in Poland to meet the minimum employment standard for hospital and company pharmacies. A study conducted in 2022 by the National Consultant for Hospital Pharmacy among managers of hospital and company pharmacies confirms this observation—as many as 51% (141/278) of the respondents stated that the minimum employment standard for pharmacists is not met in their medical entity.

The condition of the infrastructure and equipment in hospital pharmacies and hospital pharmacy departments varies. According to the NIK report, “The functioning of hospital pharmacies and hospital pharmacy departments” [17], showed that almost half of the inspected hospital pharmacies did not meet the technical requirements.

The survey conducted by the National Consultant for Hospital Pharmacy also showed that 40/278 (14.4%) of pharmacies did not meet the requirements as to the pharmacy area. Regarding the preparation of anticancer drugs, 11/85 (12.9%) of hospital pharmacies in hospitals that use these drugs did not participate in their preparation, despite the legal obligation for pharmacists to prepare cytotoxic drugs. A laboratory for the preparation of sterile drugs is owned by 61/278 (21.9%) of the surveyed pharmacies, while in 2021, as many as 137/278 (49.3%) of hospital pharmacies did not prepare a single sterile drug. With regard to the possibility of preparing drugs for parenteral nutrition, 50/278 (18%) of the surveyed pharmacies have adequate infrastructure.

The study also showed that, in terms of the distribution of medicinal products in hospitals, the dominant model is the model of departmental first aid kits, i.e., the functioning of the main pharmacy warehouse, which is a source of supplementation for smaller storage units in hospital wards. In this distribution model, full packages of drugs are issued from the pharmacy, and the individual dose for the patient is prepared by the nursing staff of the ward using the supply of drugs in the ward’s first aid kit.

The necessity to introduce changes in the functioning of the hospital pharmacy area is also indicated in state documents. The document adopted by the Council of Ministers “Zdrowa Przyszłość—Ramy strategiczne rozwoju systemu ochrony zdrowia na lata 2021–2027, z perspektywą do 2030 r”. Ref. [18] indicates a necessity for the development of hospital pharmacy infrastructure, resulting in automation, increasing the level of computerization, and reducing losses. At the same time, it is necessary to provide tools for the safe preparation of intra- and parenteral nutrition and individual treatment with cytotoxic drugs. The development of clinical pharmacy services aimed at supporting hospital teams and increasing the effectiveness of pharmacotherapy and reducing its costs should also be considered. This document also indicates the need to support the education of healthcare professions other than doctors and nurses, implemented both in the form of undergraduate as well as postgraduate and specialization training. An important issue was to encourage people to take up education in occupations related to health protection, as well as to encourage people to work in Poland. This support should concern, inter alia, pharmacists and pharmaceutical technicians.

The above-mentioned directions of changes are in line with global trends, where a hospital pharmacist gradually departs from activities related to the delivery of a medicinal product, including administrative work (e.g., purchasing procedures), towards directing their activity towards optimizing the pharmacotherapy of an individual patient and groups of patients. The trend of change was very strongly emphasized in Poland in April 2021, when the Act on the Pharmacist Profession entered into force, which significantly expands the area of activity of pharmacists, including hospital pharmacists [7]. This Act clearly indicates that a pharmacist is an independent medical profession, while emphasizing that pharmacists provide healthcare services. It points to the protection of the patient’s health and the protection of public health as the aim of practicing the profession of pharmacist. Practicing the profession of pharmacist in Poland involves:

- Exercising pharmaceutical care;
- Providing pharmaceutical services;
- Performing professional tasks;
- Performing other activities specified in the Act.

## 3. Hospital Pharmacy Solutions

The sources mentioned above indicate a uniform picture of problems and challenges in the field of pharmacy functioning in healthcare entities. The identified problems make it possible to indicate the category of support necessary for implementation in this area. The measures adopted for implementation should be primarily focused on increasing patient safety and the quality of health services provided, as well as increasing the availability of these services. We propose classifying the proposed categories of support into the following groups:

- Infrastructure, including information technology (IT);
- Optimization of work organization;
- Support for pharmaceutical staff and their development;
- Support for innovative solutions in the field of pharmacy in healthcare entities (R&D).

### 3.1. Infrastructure, including Information Technology (IT)

The infrastructure functioning in healthcare entities should ensure the maximum safety, efficiency, and ergonomics of the work. The demographic trends observed in Poland indicate the aging of the society, which, in the perspective of 2050, will mean that people aged 60 and over will constitute over 40% of the Polish population [18]. This will make it necessary to increase the expenditure on the healthcare system, as well as force an increase in its resources, including human resources. In view of the projected lower number of people of working age and the expected shortages of staff, solutions to optimize care processes supported by automation should be introduced in areas where human work can be replaced or supported by machine work.

Activities supporting the infrastructure of the pharmacotherapy area in medical entities should concern:

- Support in the development of hospital pharmacy premises, including the specification of requirements to be met by the pharmacy premises;
- Digitization of processes related to drug trading;
- Support in the development of automation.

### 3.2. Optimization of Work Organization

Ensuring access to appropriate medicinal products is a fundamental condition for ensuring patient drug safety.

Basic pharmaceutical services in relation to hospital pharmacists include organizing the supply of medicinal products, foodstuffs for nutritional uses, and certain medical devices (dressing materials, implantable devices, sterile devices for the preparation and administration of drugs, blood products, and dialysis fluids) in medical entities. The statutory duties of hospital pharmacists also include participation in the management of the indicated materials and products. The drug and medical device appropriate for the patients’ needs should be available in time when the patient’s condition requires their use.

At the same time, the area of medicinal products and medical devices management must ensure effective resource management, including inventory control, expiration dates, and rotation ratios. The analysis of warehouse management should enable substantive supervision over the use of medicinal products in accordance with the accepted standards of care.

### 3.3. Support for Pharmaceutical Staff and Their Development

Providing appropriate human resources in the field of pharmacy in medical entities is necessary to enable high-quality access to pharmaceutical services and safe pharmacotherapy. At the same time, as shown earlier, at present, there are at least 730 pharmacists’ jobs to meet the minimum standard of employment in medical entities.

The already-mentioned document “Zdrowa Przyszłość” [18] identifies the need to support professions related to healthcare, implemented both in the form of undergraduate as well as postgraduate training—improvement and specialization. A necessary element influencing the quantitative level of pharmaceutical staff is the attractiveness of the profession, including its financial attractiveness. The level of earnings of pharmacists in medical entities must correspond to the level of earnings of medical staff with similar education and competences. At the same time, the attractiveness of the profession must be increased by eliminating unnecessary bureaucratic burdens and redirecting the activity of pharmacists to the area of safety and quality of pharmacotherapy.

In the field of specialization training, it is necessary to co-finance the training from public funds. At present, the costs of specialization training are borne entirely by pharmacists. Regarding further training, educational support should be provided to hospital pharmacy personnel focused on management elements (process management and personnel management). To ensure the highest standards of functioning of Polish hospital pharmacies, it is worth considering educational support for the implementation of process management models and the standardization of activities based on quality management systems.

### 3.4. Activities in the Field of R&D

#### 3.4.1. ATMP

Advanced Therapy Medicinal Products (ATMPs) currently belong to the category of the most modern solutions in the field of widely understood pharmacotherapy. The main types of these products are cell therapy, gene therapy, and tissue therapy. At present, the popularization of these technologies is starting in Poland, particularly cell therapy technology (e.g., CAR-T) and gene therapy. In 2021, the first products of these categories were reimbursed in Poland.

ATMPs are currently manufactured in some hospital pharmacies abroad [19]. Knowledge, skills, and competence in advanced therapy medicinal products should include theoretical and practical skills in molecular and cell biology as well as the ability to use these methods for gene therapy, cell therapy, and tissue engineering. This requires training in these areas, as well as training in good manufacturing practice (GMP) for ATMP and the methodology for conducting translational research related to the challenges of hospital pharmacy [20].

The fact that this type of activity was launched in other countries means that, with appropriate preparation, products belonging to the ATMP category could also be manufactured in selected healthcare entities in Poland.

#### 3.4.2. 3D Printing

Three-dimensional (3D) printing technology can be indicated as a technology with great potential in the field of delivering individual doses of drugs, especially in pediatrics. Thanks to the use of 3D printing, it is possible not only to create a drug form in a dose precisely matched to the patient’s needs, but also to optimize the pharmacokinetics of the preparation. Innovative drug forms also allow easier dosing, e.g., the use of dispersible polymer films in children instead of powders in starch wafers or capsules [21].

Optimizing pharmacotherapy requires investment in searching for innovations and then implementing these innovations in the clinical area. The solutions proposed in this area are aimed at supporting research projects that could be tested in hospital pharmacies in the next phase (clinical trials) and, after confirming their safety and usefulness, implemented in Polish hospitals.

#### 3.4.3. Innovations in the Field of Pharmacy

The scientific resources of Polish research centers should find support among practitioners who carry out the daily tasks of hospital pharmacy. It is desirable to integrate new solutions with the environment in which they can be tested, e.g., during clinical trials. Efforts should be made to develop a framework for cooperation between research centers and Polish hospitals. Research projects should be focused on searching for innovations that would support the daily practice in the field of pharmacotherapy.

## 4. Materials and Methods

### 4.1. Data Collection and Procedures

To find out about the attitude of the hospital pharmacists, a questionnaire was constructed (see Supplementary Materials). The overriding aim of the study was to find out whether hospital pharmacists see the need to introduce changes in their profession and whether they are ready to introduce these changes. The questionnaire also included questions about the scope of the necessary changes and possible factors that may adversely affect the implementation of the proposed changes.

The questionnaire consisted of 37 questions grouped into thematic sections. The survey was prepared in electronic form using the Surveymonkey website. No sample selection was used to carry out the study. The questionnaire in the form of an open link was addressed to all hospital pharmacists in Poland. There was no selected sample because the authors wanted to reach the entire population of hospital pharmacists in Poland. The link to the survey was disseminated by the Local Pharmaceutical Chambers. It was also widespread in industry groups on Facebook and LinkedIn. The study was carried out between 26 January and 7 February 2022.

In total, 277 hospital pharmacists answered the questionnaire. There are approximately 2090 hospital pharmacists in Poland (at the end of 2020). Taking this number into account, the questionnaire was completed by approximately 13% of hospital pharmacists in Poland.

Despite the lack of a sample, the questionnaire was completed by very diverse participants (i.e., different genders, positions, public and private sector workers, from all voivodeships in Poland, different specializations, and different educational qualifications). This proves that the questionnaire reached the entire community, and its results present the opinions of the surveyed hospital pharmacists (for more details see Table 1).

### 4.2. Measures

The questionnaire had 37 questions grouped into thematic sections. The first section focusses on the sample characteristics of the participants, such as gender, position, sector, professional experience, and education level. The second section is about perceived satisfaction with the current way of practicing the profession of pharmacist, measured with a 10-item scale. The third section targets the need to introduce changes in their profession and readiness to introduce these changes. The fourth section studies the scope of the changes needed. The last section analyses the factors that may adversely affect the implementation of the proposed changes. To measure the items of corresponding variables, a standardized ten-point scale was used to organize the scale ranging from 1 (Strongly Disagree) to 10 (Strongly Agree). The questions about knowledge of clinical pharmacy services and barriers in the implementation of changes was measured with a five-item scale from 1 (No knowledge/No barrier) to 5 (Complete knowledge/Strong barrier).

### 4.3. Data Analysis

Descriptive results were generated using Microsoft Excel 2019, and IBM Corporation Statistical Package for the Social Sciences (SPSS) version 28 was used for statistical analysis (with an a priori level of statistical significance set at *p* < 0.05). The Cronbach’s alpha method (a reliability coefficient) was used in the measuring of internal consistency of the tests [22,23]. To arbitrate that there were measures of ordinal association between two possibly dependent random variables, Somers’ D was implemented [24,25]. The Pearson’s correlation coefficient with a significance level of 5% was used in the analysis of the statistical relationships and differences between the responses of the respondents with regard to the job satisfaction level and demographic characteristics of the participants [26].

## 5. Results and Discussion

Table 1 demonstrates information on the numbers of surveyed pharmacists and the assessment of the results.

Among the respondents, 165 people (59.5%) stated that they had specializations in the field of pharmacy (including 132 people with one specialization; 31 people with two specializations; two people with three specializations). Most often, the hospital pharmacists specialized in community pharmacy (43.4%). Almost a third (31.8%) specialized in hospital pharmacy, and more than a fifth (22.2%) specialized in clinical pharmacy. Only two pharmacists specialized in pharmacology.

### 5.1. Job Satisfaction

**Figure 1:**
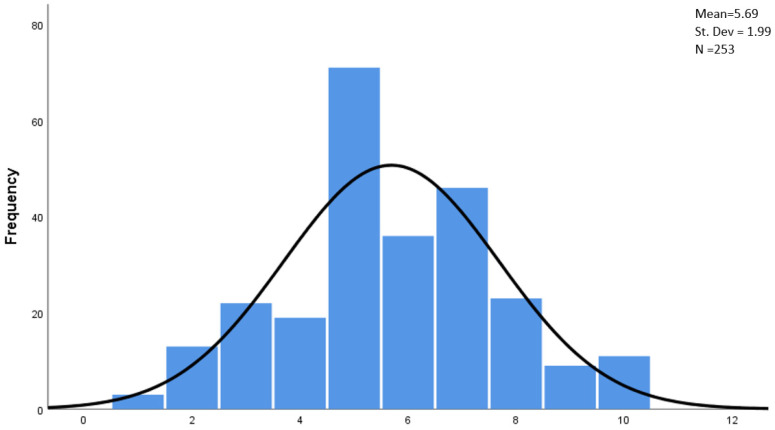
Scale of the level of satisfaction with the current way of practicing the profession of pharmacist.

The first of the main research problems was to determine the level of job satisfaction. The respondents rated this on a scale from 1 (the lowest level of satisfaction) to 10 (the highest level). The histogram for the satisfaction with the current way of practicing the profession of pharmacist is presented in Figure 1.

The level of job satisfaction defined by the respondents varies considerably. The dominant value indicated was the middle level (5), marked by 28.1% of the respondents. The extreme levels of satisfaction were indicated by relatively few pharmacists, while those with extremely low satisfaction (level 1 and 2) were slightly less (6.3%) than those with very high satisfaction (level 9–10; 7.9%).

The level of satisfaction indicated by the respondents depends on:

- The size of the hospital (*p* < 0.05, Somers’ D = 0.153)—this is a weak correlation; the smaller the facility, the more often pharmacists indicated a low or medium level of satisfaction, and the larger the entity (especially over 600 beds), the more often they indicated a high and very high level of satisfaction;
- The pharmacist’s age (*p* < 0.05; Pearson’s r = 0.177)—this is a weak correlation; the higher the age of the pharmacist, the higher the level of satisfaction;
- The number of years of work in a pharmacy (*p* < 0.05; Pearson’s r = 0.71)—this is a weak correlation; the longer the work experience, the higher the level of satisfaction.

However, the level of satisfaction is not influenced by such characteristics as gender, sector, position in a pharmacy, or having/not having a specialization.

Even though the mere possession of a specialization does not affect the level of declared satisfaction, it is worth paying attention to those pharmacists who have various types of specialization. A particularly low level was demonstrated by clinical pharmacists, among whom more than a third reported satisfaction at the level of 1–4, i.e., low and very low. At the same time, no pharmacist with this specialization indicated very high satisfaction (9–10) (Table 2).

Pharmacists with hospital and community pharmacy specializations declared a very similar level of satisfaction with their profession.

The results show some similarities, but also some significant differences, to those found in job satisfaction among the pharmacists. For example, Iorga et al. [27] found that some variables, such as age, level (pharmacist, specialist, or hospital primary pharmacist), administrative tasks (pharmacist or head pharmacist), satisfaction with the annual budget, the relationship with their colleagues, legislation, or work time revealed no significant differences. On the other hand, 86.3% of the hospital pharmacists in Romania are not at all satisfied with present legislation and 74% of subjects are dissatisfied with the annual budget. A similar conclusion was reached in another study [28,29,30,31,32,33]. They indicate that gender, job position, education level, and the location of the hospital were not significant factors in determining job satisfaction. As some studies have shown, job-related predictors of job satisfaction are skill use (as the most important factor in evaluating their ideal job) and recognition. Stavrou et al. [34] indicate factors that are directly related to the stress levels experienced by pharmacists, which can affect job satisfaction, i.e., inadequate staffing, increased responsibilities, and a high workload.

### 5.2. Need for Changes and Readiness to Make Them

Among the surveyed hospital pharmacists, 96.1% indicated the need to introduce changes to the model of functioning of pharmacists in medical entities. The few (10 respondents) who do not see such a necessity are employees of the public sector, usually over 40 years of age, without a specialization or pharmacy specialization, declaring a high and very high level of job satisfaction. However, in general, almost the entire community of hospital pharmacists indicated the need for changes.

**Figure 2:**
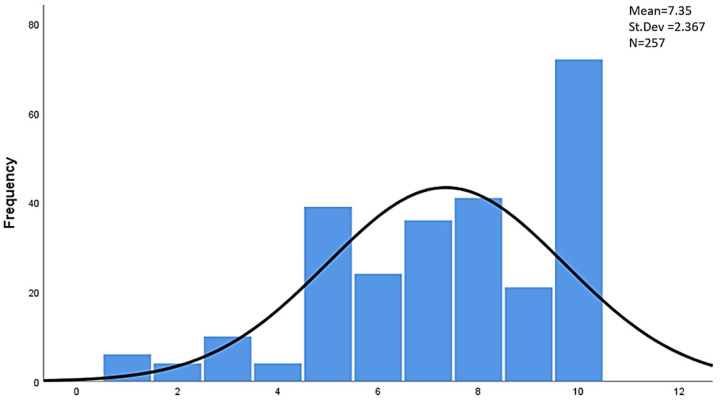
Scale of readiness to start working on change in the way pharmacists function.

The proposed changes do not only concern the general model of functioning of pharmacists in healthcare entities, but relate to its individual areas. Importantly, the need for changes in all of the areas listed in Table 3 was indicated by 59.7% of the surveyed pharmacists. Moreover, 37.4% of the respondents indicated the need for partial changes (in selected areas). Only seven respondents indicated that changes are not needed in any of the areas mentioned. At the same time, the general high level of readiness to introduce changes should be pointed out (Figure 2).

Two-thirds of the surveyed pharmacists declared high or very high readiness (levels 7–10) to start work to introduce changes. Only 9.3% of the respondents showed low or very low readiness to change (levels 1–4).

The level of readiness to introduce changes does not depend on the position in the pharmacy, gender, specialization in the field of pharmacy, the size of the entity, age, number of years of work, or even the level of job satisfaction. The only feature that was noted is that there was no pharmacist working in the private sector among the pharmacists declaring a low level of readiness to implement changes. It seems that the readiness to work on introducing changes is a feature independent of social and professional characteristics.

By analyzing the data on individual levels of readiness in the statements assessed, we can see that nearly a quarter of the respondents showed the highest readiness (level 10) to introduce changes. However, it should be emphasized that the readiness to introduce changes is a consistent attitude. The Cronbach’s alpha for the five readiness areas listed in Table 4 is equal to 0.883, which means that if a pharmacist reported a high readiness to change in one of the elements, then it is likely that in the remaining ones the declared readiness was also very high.

**Figure 3:**
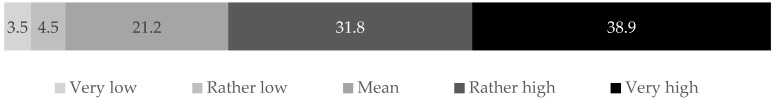
Readiness to change index.

Thus, an index of readiness to change (Figure 3) can be created (by summing up the individual degrees of readiness for each respondent and recategorizing them).

The index of readiness to introduce changes indicates that this readiness among pharmacists is very high. Only 8% of the respondents reported a low or very low level, while a very high level of readiness was demonstrated by nearly 40% of the respondents.

### 5.3. Areas of Change

#### 5.3.1. Start of Research and Development Work

The need to conduct research and development (R&D) work in hospital pharmacies was reported by 82.4% of the respondents, and the average readiness to implement them was 6.83. It was both the least frequently supported need to introduce changes in hospital pharmacies, as well as the lowest level of readiness to introduce them (compared to other areas of changes).

The pharmacists indicated that in terms of changes in the R&D area, the most important thing is participation in the clinical trials of medicinal products and medical devices. The absolute necessity of including hospital pharmacies in these activities was indicated by 59.5% and 48.2% of the respondents, respectively, and the idea itself was supported by approximately three-quarters of the respondents.

Research in innovative medication forms and dose individualization as well as the preparation of innovative medicinal products in hospital pharmacies met with much lower support. Every fourth respondent supported the absolute need to introduce them, and the idea itself was supported by slightly more than half of the respondents.

**Figure 4:**
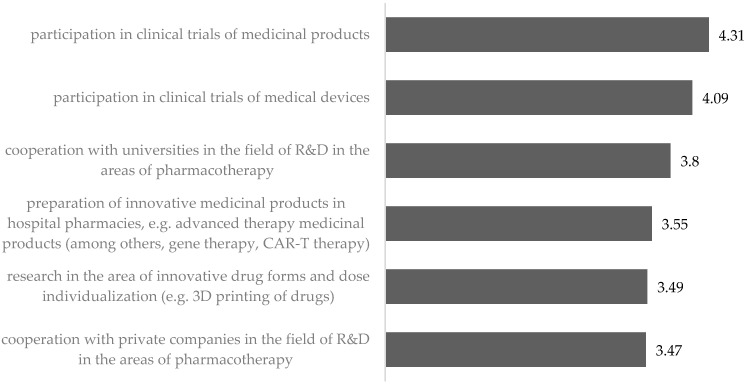
Assessment of the need to introduce changes in the area of research and development—mean.

It is worth noting that pharmacists more willingly see universities as partners to cooperate in the field of pharmacotherapy than private companies (Figure 4).

When pointing to the possibility of starting R&D work, the pharmacists were asked who would pay for such research. Among the submitted opinions, it was emphasized that the introduction of this type of solution requires, above all, the good education of pharmacists and specific regulation of the role of pharmacists in clinical trials/research/tests. Within this area, three statements are important:

*“Greater direct involvement of pharmacists (not only in the preparation of medications and their storage), but active participation in the collection of data and their analysis”* (R185)

*“Equipping pharmacies with laboratories for testing the durability and quality of medications prepared—HPLC, HPLC-MS, IR spectra”* (R180)

*“A clear indication that if the clinical trial entity is located in the same location as a hospital pharmacy, the medications must be registered with the pharmacy and additional funding is necessary for the pharmacist as a member of the research team”* (R66)

Therefore, launching work in the R&D area in hospital pharmacies requires, on the one hand, the good preparation of pharmacists themselves, and on the other hand, a clear definition of the scope of their involvement in R&D work.

In Poland, the law does not specify the role of pharmacists in conducting clinical trials in detail. The Act on the pharmacist profession adopted in 2021 mentions participation in clinical trials, including trials conducted in a hospital as a member of a research team, among the professional tasks of a pharmacist. The Act indicates the participation of pharmacists in conducting clinical trials, suggesting that a pharmacist may, in part of the research, be a member of the research team. It is legitimate for the pharmacist to be a part of the research team in those clinical trials where the pharmacist must prepare or participate in the preparation of the investigational medicinal product. In some clinical trials, the pharmacist’s participation is very important, particularly in trials where cytotoxic drugs are prepared [35]. Clinical trials are defined as trials in which patients participate and the purpose of which is to determine the safety and efficacy of a new form of treatment or prevention of a given disease [36]. They are an essential element in the development of medicine [37,38]. Clinical trials, as a fundamental stage in the development of new drugs, are inherently associated with hospitals, because they often play the role of dynamic and large centers of clinical trials. In large facilities such as hospitals, apart from the Principal Investigator and their team, a hospital pharmacist is indispensable; hence, every pharmacist working in hospital facilities should be aware of how this topic is related to them.

A hospital pharmacist may be a member of the clinical trial team at a research site. Regulska [39] indicates that the pharmacist in the research team plays the role of a specialist in matters related to the investigational drug and comparator. The work of a pharmacist in clinical trials is not limited to registering drugs. In addition to receiving, storing, and dispensing the test drugs, the pharmacist prepares the preparations in accordance with the test protocol, keeps the documentation necessary for their preparation, and is in constant contact with the team members.

Increasingly, hospitals are implementing internal procedures by which clinical trials are conducted. Therefore, the Clinical Research Coordinator (CRC), the pharmacist responsible for the documentation of the research conducted at the hospital and the hospital pharmacy, is more and more often responsible for clinical trials, with information about the main researchers, the pharmacists who are part of the research team, the investigated drug, and the ward that participates in each study [40].

#### 5.3.2. Changes in the Way Pharmacists Operate in Distribution of Medicinal Products and Medical Devices

The need to introduce changes in this area was reported by 83.5% of the respondents, and the average readiness to introduce them was 7.11. In terms of changes in the distribution of medicinal products and medical devices, pharmacists identified four main needs:

- Communication (digitization of processes; medication tracking systems);
- Regulatory (development of nationwide standards regulating the rules of conduct);
- Management (educational programs in the field of quality management);
- Technical (new requirements for pharmacy premises).

**Figure 5:**
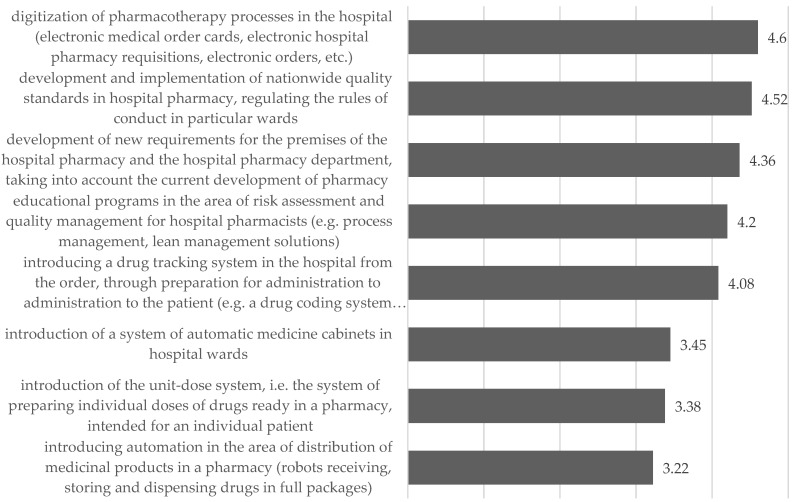
Assessment of the need to introduce changes in the distribution area—mean.

The pharmacists assessed the need to introduce automation in the distribution area as being significantly lower. At the same time, they indicated that the unit-dose system is not adapted to the needs of pediatrics (Figure 5).

Among the submitted comments, an important indication is that the pharmacist should not deal with the distribution process at all, and this task should be performed by specialized pharmacy technicians. The pharmacist should focus on the clinical aspects. The pharmacists indicated that they are burdened with a huge amount of administrative work (tenders and settlements) and that their knowledge and qualifications are not used for the benefit of patients, but more for bills and statistics. The respondents also pointed to the resistance of the medical community in the use of hospital systems to conduct electronic orders, which has a limiting effect on the digitization of the system.

Although Information and Communication Technology (ICT) transforms many sectors, the health sector is lagging in adopting ICT. In the manual system, much of the data are difficult to access and are not available in real time. There is lack of coordination between clinical service providers (doctor, nurse, patient, and management) and other services such as pharmacy, procurement, laboratory, and radiology. The digitization of the hospital is vital in providing quality and cost-effective services to patients and improving support services [41]. Advances in the application of artificial intelligence, digitization, technology, cloud computing, and wearable devices in healthcare are the future for healthcare professionals and patients. Some researchers [42] recommend a structured approach to prepare healthcare professionals and patients for emerging pharmacotherapy needs. Firstly, clinician training should include genomics, cloud computing, the use of large datasets, implementation science, and cultural competence. Secondly, patients will need support for wearable devices and reassurance regarding digital medicine.

Health system pharmacists, like other healthcare professionals, practice under several mandated standards. The basic concepts of quality assurance (QA) standards should be applied to hospital pharmacy practice [43]. Many countries have implemented national quality standards in hospital pharmacy, regulating the rules of conduct in individual wards [44,45].

#### 5.3.3. Changes in Preparation of Medicinal Products

The need to introduce changes in this area was reported by 85.9% of the respondents, and the average readiness to introduce them was 7.11.

The pharmacists indicated that in terms of changes in the preparation of medicinal products, the most important thing is to make it possible to settle the excess of directly financed medications with the National Health Fund and the introduction of a taxa laborum fee for the preparation of sterile medicinal products in hospital pharmacies. The absolute necessity to include hospital pharmacies in these activities was indicated by, respectively, 61.2% and 51.5% of the respondents, and the idea itself was supported by approximately ¾ of the respondents.

**Figure 6:**
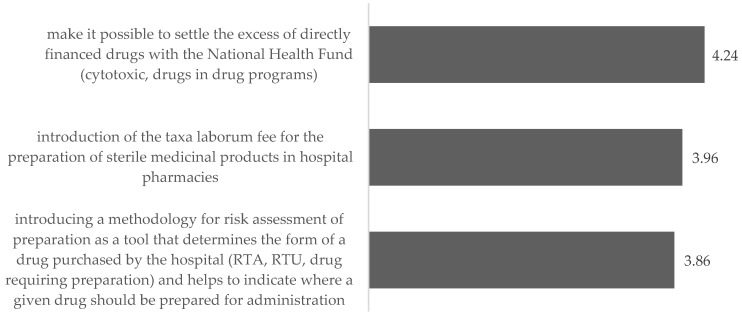
Assessment of the need to introduce changes in preparation of medicinal products—mean.

The introduction of a methodology for the risk assessment of preparation as a tool that determines the form of a medication purchased by the hospital and helps identify where a medication should be prepared for administration has received much less support. Every third respondent supported the absolute need to introduce it, and the idea itself was supported by slightly more than half of the respondents (Figure 6).

Two threads dominate in the statements of pharmacists: the first concerns the need to introduce specific organizational regulations and the second relates directly to the preparation of medicinal products.

The regulatory aspect is well presented by the following statement:

*“Guaranteeing, by means of a decree of the Minister of Health, appropriate local and staffing standards and the necessary equipment at a level that allows the preparation of medications in accordance with the current guidelines and standards; developing solutions facilitating the centralization of medication preparation for several entities in one place in order to optimize the management of medicinal products, reduce medication losses and guarantee the highest standards of medication preparation”*. (R4)

Pharmacists emphasize staff shortages and the lack of up-to-date regulations regarding the requirements that pharmacies must meet. At the same time, they indicate that the Pharmaceutical Inspection does not understand the specificity of the preparation of medications and “does not keep up” with the progress in this field. Therefore, a proposal was made for practical training for supervisory authorities. The aspect of medicinal products focused on the needs related to cytotoxic medications (costs of production losses, disposal of residues), preparation of parenteral nutrition preparations (certification of premises), support in the preparation of parenteral medications (appropriate computer programs eliminating the risk of confusion), and the development of prescription medication standards.

The preparation of medicines in pharmacies is important to accommodate individual patients’ needs in Europe. This is the case if an appropriate authorized medicine does not exist or is unavailable on the market [46]. However, some aspects of pharmacy preparations, notably the standards for quality assurance and safety, are not harmonized throughout Europe and therefore fall under the competencies of individual member states. Some authors [47] investigate the suitability of good manufacturing practice (GMP) for the quality assurance of the preparation in hospital pharmacies. The results obtained by the other researchers show that the expansion of GMP with quality by design (QbD) and quality risk management (QRM) significantly improve the applicability for all types of preparation in hospital pharmacies. QbD and QRM acknowledge the importance of targeting the needs of the patient, of a good design, and of risk assessment, thereby offering more flexibility to the hospital pharmacist to respond to everyday requests for patient care.

#### 5.3.4. Changes in the Way Pharmacists Operate in Hospitals by Launching a Clinical Pharmacy Service

The need to introduce changes in this area was reported by 95.4% of the respondents and it was the need for changes most often reported by pharmacists. The average readiness to introduce them was 7.05.

The pharmacists indicated that in terms of changes in the way pharmacists operate in hospitals by launching a clinical pharmacy service, the important things are almost all areas except one, i.e., issuing follow-up prescriptions. The most important thing is medication review (MR), creating a hospital antibiotic policy, and education in the field of pharmacotherapy aimed at patients or medical personnel. The absolute necessity to include hospital pharmacies in these activities was indicated by 58.4%, 55.3%, and 54.8% of the respondents, respectively, and the idea itself was supported by approximately 80% of the respondents.

**Figure 7:**
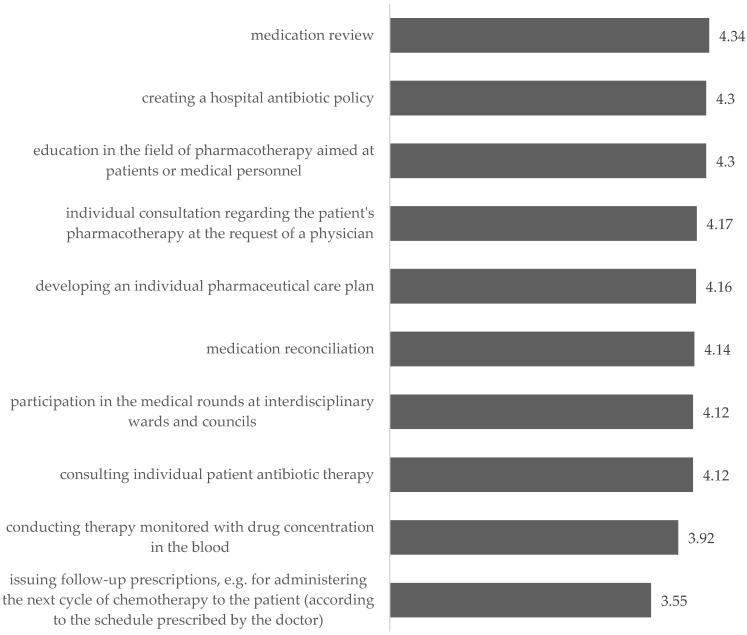
Assessment of the need to introduce clinical pharmacy services—mean.

The individual consultation regarding the patient’s pharmacotherapy at the request of a physician, developing an individual pharmaceutical care plan, and medication reconciliation received a little less support. Almost every second respondent supported the absolute need to introduce them, and the idea itself was supported by slightly more than half of the respondents (Figure 7).

The pharmacists stressed that a clinical pharmacist should be employed in a separate team that will provide services to the entire hospital. This form of employment was indicated by 50.9% of the respondents. In addition, 38% stated that a clinical pharmacist may be employed in a hospital pharmacy, but only on the condition that a team of clinical pharmacists is designated. The idea of employment directly in a ward or in a hospital pharmacy under the supervision of the manager did not find much support among pharmacists (these options were chosen by 5.6% of the respondents).

The respondents emphasized that the introduction of clinical pharmacy services requires the training of both pharmacists and medical staff. This service should also include:

- Forms of patient education (especially patients qualifying for transplant);
- Running a medication information center;
- Monitoring of undesirable effects, analysis of complications and treatment failure, and analysis of prolongation of hospitalization;
- Pharmacotherapy supervision after discharge from the hospital.

Changing social needs means that pharmacists are faced with new challenges, forcing them to specialize in a clinical direction [48,49]. According to the Supreme Pharmaceutical Chamber in Poland and the Polish Pharmaceutical Society [50], clinical pharmacy is defined as:

*“(…) targeted teaching of pharmacy students and practicing the profession of pharmacist for the safe and proper use of a medicinal product in a patient, thus placing the main emphasis on the importance of a medicinal product for the patient in the entire complexity of this issue, including the route of administration, form and formulation of the medicinal product, dosage, interactions with other medicinal products, food, and diagnostic tests”.*

Throughout the world, this concept is defined and interpreted in various ways. The most frequently cited definition is the American College of Clinical Pharmacy (ACCP). The ACCP defines clinical pharmacy as *“the area of pharmacy encompassing scientific knowledge and the practice of rational drug use”* [51].

The potential benefits of clinical pharmacy have long been recognized in the US, Canada, Australia, the UK, France, and many other countries [52,53,54,55,56]. For instance, in Germany, because of the small number of hospital pharmacists, the clinical service is targeted to wards with intensive drug therapy (e.g., intensive care units and oncology units), or wards with an increased need for drug therapy consultations (e.g., surgery wards). In addition, the service is not necessarily offered daily [57]. In Poland, the profession of clinical pharmacist is just developing, but in the UK, doctors cannot imagine working in a hospital ward without a clinical pharmacist. This means great comfort and safety for them. Their cooperation is aimed at providing the most effective help to the patient. The doctor is a specialist in diagnosis and treatment. On the other hand, a clinical pharmacist advises on the selection of the appropriate medication or the method of its administration.

Medication review (MR) is an integral part of a clinical pharmacist’s interventions [58]. MR is undertaken to identify and reduce medication errors and to optimize the treatment of the patient [51]. MR is defined as “a structured, critical examination of a patient’s medicines with the objective of reaching an agreement with the patient about treatment, optimizing the impact of medicines, minimizing the number of medication-related problems and reducing waste” [59]. Increasing drug consumption together with patient metabolic changes with age predisposes older people to medication-related problems, e.g., reduced elimination due to renal impairment and ADEs [60,61].

Medication reconciliation is the process of creating the most accurate list possible of all medications a patient is taking (i.e., drug name, dosage, frequency, and route, and comparing that list against the physician’s admission, transfer, and/or discharge orders) with the goal of providing correct medications to the patient at all transition points within the hospital [62]. Previous studies have shown the importance of undertaking MR as a means of identifying medication-related problems and thus positively contributing to patient care [52,55,63].

### 5.4. Problems Related to Clinical Pharmacy Implementation

**Figure 8:**
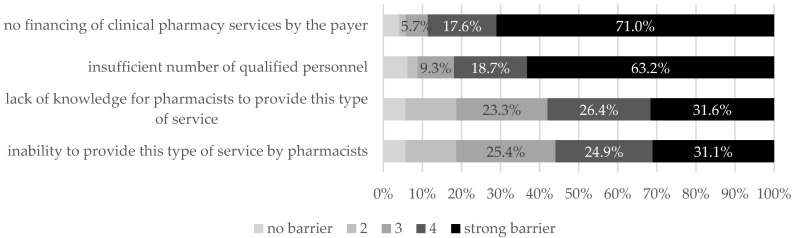
Assessment of obstacles to the introduction of the clinical pharmacy service.

Regarding the main obstacles to the introduction of a new service, the pharmacists cited a lack of funding and a lack of sufficient personnel. However, the next questions of the survey indicated that the knowledge and skills possessed by hospital pharmacists are no less important problems. Although more than half of the respondents indicated that the lack of knowledge and skills to provide clinical pharmacy services is an obstacle to launching the service, when asked directly about the assessment of their knowledge and skills in conducting clinical pharmacy, only 30% of the respondents indicated that they have such knowledge and skills (Figure 8).

**Figure 9:**
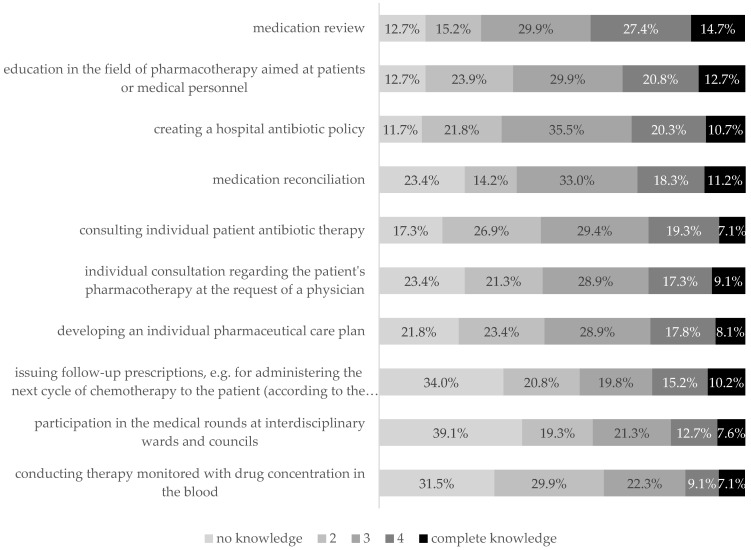
Knowledge of the individual tools of clinical pharmacy.

Detailed information on the knowledge of pharmacists in the field of clinical pharmacy is provided by the question in which the respondents assessed their own knowledge of clinical pharmacy tools (Figure 9). In fact, only in the case of three tools (medication review, education in the field of pharmacotherapy addressed to patients or medical personnel, and creating hospital antibiotic policy) was the declared level of knowledge higher than the declared lack of knowledge of the tools.

The number of staff as well as knowledge and skills appear to be the most significant obstacle. In the open statements, the pharmacists emphasized the problems related to:

- The opportunity to specialize:

*“Very, very difficult access to specialization in both hospital and clinical pharmacy. A very small number of places, even though the specialization is payable about PLN 10,000. There are only 3 training centers for the whole of Poland”.* (R145)

*“No (co)funding from hospitals for pharmacists wishing to deepen their knowledge. No possibility of periodic training and educational leaves”.* (R158)

- The opportunity to gain experience:

*“Currently, the very limited presence of pharmacists in the field of even the basic clinical aspects of the work of hospital wards, which causes a lack of understanding of people working there (doctors/physicians, nurses) of the need to involve pharmacists in this area. It is necessary to involve pharmacists on the largest possible scale, initially primarily in areas not implemented or reluctantly implemented by other professional groups (e.g., medication conciliation, medication reviews, patient, and staff education) to broadly demonstrate the benefits of involving pharmacists in the process of therapy and care of patient. Consequently, this is leading to the natural willingness of doctors and nurses to involve more pharmacists at the higher levels of clinical pharmacy services and to build confidence in such changes”.* (R4)

*“Lack of clinical experience—necessary support of medical staff, no specialization in a given field of medicine, e.g., oncology and pediatric hematology, science takes a lot of private time, no defined responsibilities, competences, and powers of a clinical pharmacist”.* (R248)

An additional obstacle is the fear of more work in connection with the launch of new clinical activities for pharmacists, which was reported by as many as 70.5% of the respondents. This concern, combined with the information that only 15.7% of respondents have time to start and provide a new service, indicates that the introduction of the service requires a reorganization of the responsibilities of hospital pharmacists. As one respondent pointed out:

*“Due to the shortage of staff and the number of tasks in the activities of the basic hospital pharmacy, the chances of delegating a pharmacist strictly to work in the ward are almost zero”.* (R206)

The last, but equally important obstacle as mentioned above is the lack of organizational preparation. Only 7.4% of the surveyed pharmacists admitted having the necessary tools (procedures, instructions, questionnaires, etc.) to start and provide clinical pharmacy services.

Among other barriers, pharmacists mentioned: a lack of awareness of people managing the medical entity; non-acceptance by medical staff and nurses; and hardware and IT barriers (no access to data). In the opinions expressed, the role of the pharmacist in the hospital is rather underestimated. This is because pharmacy masters (of whom there are too few) deal with organizational matters.

*“Still a big problem is the lack of financing for services and too few masters who deal with, for example, tenders daily (because it is important for the Directorate). Without funding, it will be difficult to get through. The hospital pharmacy must start earning money so that it is profitable and noticeable in the eyes of the management in the way we wish”.* (R153)

Additionally, the role of other pharmacists is perceived in a marginal way. According to some, the pharmacist is shifting cardboard boxes and *“dragging carts with equipment to the wards”* (R183).

Hospital pharmacists (HPhs) recognize the importance of clinical pharmacy in their current practice. However, the biggest barriers they face are lack of financing, insufficient number of staff, lack of knowledge and skills, workload (lack of time, fear of additional duties), lack of tools to use the service (procedures, instructions), and awareness of hospital managers. Further training may be useful to ensure HPhs are adequately prepared to undertake clinical activities.

The main issue in clinical pharmacy implementation is to identify the source and method of financing, which has not been specified in the Act [7]. Healthcare in Poland is mainly financed by public sector entities, among them being the National Health Fund (NFZ), the state budget, and local government budgets. The task of the National Health Fund, as the main payer in the system, is chiefly currently financing the services [64]. Some pharmacies, even without additional funds, are ready to make some of the components able to use the competences of pharmacists. Research shows that the preferred source of financing by society is paying with public funds. Perhaps funding should be based on a mixed system, with part of the services covered by the public system and part by direct patient payments or additional insurance.

The first research limitation is related to the national policies and legislation that are applied in this medical field, which have an important impact on the level of satisfaction among hospital pharmacists in Poland and its expression needs to change; the second limitation is due to the short period the conducting of research, which, in turn, used a small sample. The small sample size potentially limits the generalizability of the sample to hospital pharmacists in Poland. Finally, another limitation is due to the fact that no sample selection was used. The questionnaire was addressed to all possible persons in Poland to reach the widest possible group of hospital pharmacists. Although this is a limitation, it does not affect the quality of the study. We have studied HPhs from private and public hospitals, from all regions in Poland, and in different specializations.

## 6. Conclusions

Europe needs intelligent investments in healthcare, new ways of implementing innovations, and smart and reasonable restructuring of the healthcare system, otherwise the accessibility to healthcare systems in many societies may be a challenge. The introduction of the long-awaited regulation into the Polish legal system made it possible to use the professional potential of pharmacists and pharmacies more effectively, created legal opportunities for additional pharmaceutical services for patients, and ensured the independence and professional independence of pharmacists. Regulation is also particularly important in the face of health challenges faced by the healthcare system. The growing number of elderly patients and the gradually decreasing number of doctors and nurses, the COVID-19 pandemic, and the ongoing war on the territory of Ukraine make it necessary to continue to implement new solutions focused on extending the powers of pharmacists, the scope of which is clearly defined by the Act.

The results of the study show that the amount of work of a non-medical nature causes frustration and fatigue among pharmacists, causes lack of professional performance, and even becomes the cause of occupational burnout. Hospital pharmacists overwhelmingly indicated the necessity to introduce changes in each area studied, while demonstrating a high degree of readiness to undertake work on the implementation of new solutions.

The obtained results of the study leave no doubt that the community of hospital pharmacists in Poland expects real changes in the way of practicing their profession. The necessity of change is apparent among people working in this profession. Pharmacists are ready for further, dynamic, and wisely introduced systemic changes that will strengthen their position in the system and translate into the effectiveness and safety of patients’ pharmacotherapy.

Clinical pharmacy specialists feel the least satisfied with the current way of performing their profession, at the same time declaring one of the highest levels of readiness to undertake change. This may be because clinical pharmacists are highly motivated people who want to develop and use the acquired clinical skills, but at present, they have little opportunity to fulfill themselves in this area.

Although hospital pharmacists recognize the importance of clinical pharmacy in their current practice, the biggest barriers are the lack of sufficient pharmacists to provide clinical pharmacy services, as well as deficiencies in the education, skills, and tools necessary to deliver them, along with a lack of funding. Ensuring an appropriate level of medical staff will undoubtedly be one of the major challenges for the healthcare system in the coming years. A solution to the problem of staff shortages in the field of hospital pharmacy could come from the fields of digitization, automation, and robotization of this area. While pharmacists’ acceptance of digital solutions is high, automated solutions and robots are less popular. In our opinion, it is necessary to introduce solutions that will redirect pharmacists from non-medical areas to the clinical sphere of their profession. At the same time, one should not forget about the need to provide adequate hospital pharmacists, which is necessary to maintain the continuity of supplying patients with individual doses of medicinal products, which are prepared in hospital pharmacies.

These results are important for hospital pharmacists and hospital management to focus on healthcare policies, management, and environmental issues, with the purpose of improving the existing model of the profession of hospital pharmacist. Knowledge regarding job satisfaction will enable employers to respond to employees’ needs, decrease turnover, and improve the work environment.

In further research, the approach will continue by, for example, focusing on the role of hospital pharmacists and a study could be conducted in other medical professional groups because hospital pharmacists operate in the healthcare system, which also covers other medical professional groups, such as physicians and nurses.

## Figures and Tables

**Table 1:** Demographic characteristics of participants (*n* = 277).

Demographic Variables	Description	*n* (%)
Gender	Female	207 (74.8%)
	Male	70 (25.2%)
Position	Pharmacy manager in public hospital	127 (46%)
	Pharmacist	122 (44.2%)
	Pharmacy manager in private hospital	17 (6.2%)
	Other including pharmacy owner or partner	10 (3.6%)
Sector	Pharmacist working in public sector	253 (91.2%)
	Pharmacist working in private sector	24 (8.8%)
Year of working experience	Up to 5 years	36 (13%)
	Between 6 and 10 years	49 (17.7%)
	Between 11 and 20 years	95 (34.3%)
	Over 21 years	97 (35%)
Size of hospital	Up to 100 beds	28 (10.1%)
	Between 101 and 300 beds	103 (37.2%)
	Between 301 and 600 beds	81 (29.2%)
	Between 601 and 1000 beds	42 (15.2%)
	Over 1000 beds	23 (8.3%)
Educational qualification	Master of Pharmacy (MPharm)	261 (93.9%)
	PhD in Pharmaceutical Sciences (PSC)	17 (6.1%)

Source: Primary data.

**Table 2:** Satisfaction with the current way of practicing the profession of pharmacist and specialization.

Job Satisfaction	No Specialization	Specialization		
		Hospital Pharmacy	Clinical Pharmacy	Community Pharmacy
Very low (1–2)	6.5%	8.3%	7.1%	3.8%
Low (3–4)	17.0%	11.7%	28.6%	12.5%
Medium (5–6)	37.9%	30.0%	47.6%	36.2%
High (7–8)	30.1%	36.7%	16.7%	35.0%
Very high (9–10)	8.5%	13.3%	0.0%	12.5%
Overall (N)	100	60	42	80

**Table 3:** Need for change implementation.

Need to Make Changes	Yes (%)	N
In the model of functioning of pharmacists in medical entities	96.1	259
In the way pharmacists operate in distribution of medicinal products and medical devices	83.5	243
In preparation of medicinal products	85.9	227
In the way pharmacists operate in a hospital by launching a clinical pharmacy service	95.4	216
Launching R&D work (in cooperation with a university or a private company) in the areas of medication form technology, new medication technologies	82.4	222

**Table 4:** Readiness to make changes.

Please Rate on a Scale of 1–10 Your General Readiness to Start Work On:	1	2	3	4	5	6	7	8	9	10	Overall	
	(%)										Mean	SD
Making changes to the way pharmacists operate in your hospital	2.3	1.6	3.9	1.6	15.2	9.3	14.0	16.0	8.2	28.0	7.35	2.37
Introducing new solutions in the distribution area	3.8	0.4	3.8	2.5	17.6	10.0	13.8	16.3	8.4	23.4	7.11	2.39
Introducing new solutions in the field of preparation of medicinal products	4.1	1.8	1.4	4.6	18.7	10.0	11.0	14.2	5.9	28.3	7.11	2.52
Introducing new solutions in R&D	4.7	5.1	3.3	5.1	15.4	7.0	11.7	16.4	7.0	24.3	6.83	2.70
Starting work on the launch of clinical pharmacy services in your hospital	4.9	1.9	3.4	7.3	15.0	7.8	9.2	12.6	8.7	29.1	7.05	2.69

## Data Availability

Not applicable.

## Supplementary Materials

The following supporting information can be downloaded at: https://www.mdpi.com/article/10.3390/ijerph192114522/s1. Questionnaire: Questionnaire_ Surveymonkey in Polish and in English.

## References

[1] FIP https://fiphsa.com/docs/assessments/fip-basel-statements-en.pdf.

[2] Bragazzi N.L., Mansour M., Bonsignore A., Ciliberti R. (2020). The Role of Hospital and Community Pharmacists in the Management of COVID-19: Towards an Expanded Definition of the Roles, Responsibilities, and Duties of the Pharmacist. Pharmacy.

[3] Yfantopoulos N., Yfantopoulos P., Yfantopoulos J. (2016). Pharmaceutical Policies under Economic Crisis: The Greek case Authors. JHPOR.

[4] Goodrick E., Reay T. (2011). Constellations of Institutional Logics: Changes in the Professional Work of Pharmacists. Work. Occup..

[5] Farmaceuta w Polsce (2019). Ogólnopolskie Badania Wizerunkowe.

[6] Deloitte (2018). Jak Wprowadzić w Polsce Opiekę Farmaceutyczną.

[7] Ustawa z Dnia 10 Grudnia 2020 r. o Zawodzie Farmaceuty (Dz.U. 2021 Poz. 97). (In English: The Act on the Pharmacist Profession). https://isap.sejm.gov.pl/isap.nsf/download.xsp/WDU20210000097/U/D20210097Lj.pdf.

[8] Sørensen K., Pelikan J.M., Röthlin F., Ganahl K., Slonska Z., Doyle G., Fullam J., Kondilis B., Agrafiotis D., Uiters E. (2015). Health literacy in Europe: Comparative results of the European health literacy survey (HLS-EU). Eur. J. Public Health.

[9] Merks P., Świeczkowski D., Kazimierczak J., Białoszewska K., Olszewska A., Krysiński J. (2015). Apteka szpitalna jako prawdziwe miejsce sprawowania opieki farmaceutycznej. Nauka Praktyka. Czas. Aptek..

[10] Żak K. (2018). Opieka farmaceutyczna czy profesjonalne doradztwo? Bariery wdrażania opieki farmaceutycznej w Polsce. Ekon.—Wroc. Econ. Rev..

[11] Ayalew M., Taye K., Asfaw D., Lemma B., Dadi F., Solomon H., Tazeze H., Tsega B. (2017). Patients’/clients’ expectation toward and satisfaction from pharmacy services. J. Res. Pharm. Pract..

[12] Berenguer B., La Casa C., de la Matta M.J., Martin-Calero M.J. (2005). Pharmaceutical Care: Past, Present and Future. Curr. Pharm. Des..

[13] Gupchup G.V., Singhal P.K., Dole E.J., Lively B.T. (1998). Burnout in a sample of HMO pharmacists using the Maslach Burnout Inventory. J. Manag. Care Pharm..

[14] Nelson D.L., Simmons B.L., In J.C., Quick L.E., Tetrick (2003). Health Psychology and Work Stress: A More Positive Approach. Handbook of Occupational Health Psychology.

[15] (2020). Opieka Farmaceutyczna. Kompleksowa Analiza Procesu Wdrożenia.

[16] Rejestr Aptek (a database Register of Pharmacies including data of all pharmacies registered in Poland). https://rejestrymedyczne.ezdrowie.gov.pl/ra/search/public.

[17] NIK (2018). Funkcjonowanie Aptek Szpitalnych i Działów Farmacji Szpitalnej.

[18] Uchwała nr 196/2021 Rady Ministrów z Dnia 27 Grudnia 2021, Zdrowa Przyszłość. Ramy Strategiczne Rozwoju Systemu Ochrony Zdrowia Na Lata 2021–2027, z Perspektywą do 2030 r. Ministerstwo Zdrowia. https://www.gov.pl/attachment/4a9bd160-e052-4a52-8fd4-b7c546d556f8.

[19] De Goede A. Presentation, Hospital pharmacists involved in APTM and in Risk Assessment. Proceedings of the 23rd Congress of the EAHP.

[20] Guiu J.M. (2014). Advancing Hospital Pharmacy Practice through New Competences in Advanced Therapy Medicinal Products. Am. J. Pharm. Educ..

[21] Stępniak P., Bialik W., Żuk A. (2017). Systemy automatycznej dystrybucji leków (unit dose) w szpitalach w Polsce oraz w wybranych krajach świata. Ann. Acad. Med. Silesiensis.

[22] Cronbach L.J. (1951). Coefficient alpha and the internal structure of tests. Psychometrika.

[23] Cho E. (2016). Making Reliability Reliable. Organ. Res. Methods.

[24] Somers R.H. (1962). A new asymmetric measure of association for ordinal variables. Am. Sociol. Rev..

[25] Newson R. (2002). Parameters behind “nonparametric” statistics: Kendall’s tau, Somers’ D and median differences. Stata J..

[26] (1988). Rodgers; Nicewander, Thirteen ways to look at the correlation coefficient. Am. Stat..

[27] Iorga M., Dondaș C., Soponaru C., Antofie I. (2017). Determinants of Hospital Pharmacists’ Job Satisfaction in Romanian Hospitals. Pharmacy.

[28] Carvajal M.J., Popovici I., Hardigan P.C. (2021). Gender and Pharmacists’ Career Satisfaction in the United States. Pharmacy.

[29] Mattsson S., Gustafsson M. (2020). Job Satisfaction among Swedish Pharmacists. Pharmacy.

[30] Chou Y.-C., Dang V.T., Yen H.-Y., Lai K.-M. (2019). Influence of Risk of Drug–Drug Interactions and Time Availability on Patient Trust, Satisfaction, and Cooperation with Clinical Pharmacists. Int. J. Environ. Res. Public Health.

[31] Policarpo V., Romano S., António J.H.C., Correia T.S., Costa S. (2019). A new model for pharmacies? Insights from a quantitative study regarding the public’s perceptions. BMC Health Serv. Res..

[32] Liu C.S., White L. (2011). Key determinants of hospital pharmacy staff’s job satisfaction. Res. Soc. Adm. Pharm..

[33] Sansgiry S.S., Ngo C. (2003). Factors affecting job satisfaction among hospital pharmacists. Hosp. Pharm..

[34] Stavrou G., Siskou O.C., Talias M.A., Galanis P. (2022). Assessing Job Satisfaction and Stress among Pharmacists in Cyprus. Pharmacy.

[35] Zięcik P., Miłowska K. O Roli Farmaceuty w Prowadzeniu Badań Klinicznych—Wybrane Zagadnienia. https://zflegal.pl/aktualnosci/o-roli-farmaceuty-w-prowadzeniu-badan-klinicznych-wybrane-zagadnienia.

[36] Learn About Clinical Studies—ClinicalTrials.gov. https://clinicaltrials.gov/ct2/about-studies/learn.

[37] Dlaczego Prowadzi Się Badania Kliniczne. https://www.badaniaklinicznewpolsce.pl/o-badaniach-klinicznych/podstawowe-informacje/dlaczego-prowadzi-sie-badania-kliniczne/.

[38] Regulation (EU) No 536/2014 of the European Parliament and of the Council of 16th April 2014 on Clinical Trials on Medicinal Products for Human Use, and Repealing Directive 2001/20/EC. Dz.U. L 158 z 27.5.2014. https://eur-lex.europa.eu/legal-content/EN/TXT/PDF/?uri=CELEX:32014R0536&qid=1667573910313&from=EN.

[39] Regulska K. Leki Innowacyjne w Badaniach Klinicznych. https://www.woia.pl/news/3346/leki-innowacyjne-w-badaniach-klinicznych.html.

[40] Chudziński W. Badania Kliniczne w Aptece Szpitalnej, Aptekaszpitalna.pl. https://www.aptekaszpitalna.pl/farmaceuta-w-szpitalu/badania-kliniczne-w-aptece-szpitalnej/2018-11-02.

[41] Bele N., Kumar S., Singay J. (2018). Conceptual Framework of Digitization of Hospital Services and Operations. Int. J. Health Edu. Med. Inf..

[42] Zarowitz B.J. (2020). Emerging Pharmacotherapy and Health Care Needs of Patients in the Age of Artificial Intelligence and Digitalization. Ann. Pharm..

[43] Dargahi H., Khosravi S.H. (2010). Hospitals pharmacy quality assurance system assessment in tehran university of medical sciences, iran. Iran. J. Public Health.

[44] American Society of Health-System Pharmacists (2013). ASHP guidelines: Minimum standard for pharmacies in hospitals. Am. J. Health-Syst. Pharm..

[45] Piction C. (2014). Professional Standards for Hospital Pharmacy Services. Optimising Patient Outcomes from Medicines. England, Scotland and Wales.

[46] Scheepers H.P., Langedijk J., Handlos V.N., Walser S., Schutjens M.H., Neef C. (2017). Legislation on the preparation of medicinal products in European pharmacies and the Council of Europe Resolution. Eur. J. Hosp. Pharm..

[47] Bouwman Y. (2012). Andersen, LMGMP and preparation in hospital pharmacies. Eur. J. Hosp. Pharm. Sci. Pract..

[48] Piecuch A., Kozłowska-Wojciechowska M., Jaszewska E., Makarewicz-Wujec M. (2014). Farmaceuta kliniczny—Odpowiedź na zmieniające się potrzeby społeczne (In English: Clinical pharmacist—A response to changing social need). Farm. Pol..

[49] WHO/FIP (2006). Developing Pharmacy Practice. A Focus on Patient Care Handbook—2006 Edition.

[50] NIA/PTFarm (2007). Strategia wdrażania opieki farmaceutycznej w Polsce. Biul. Nacz. Rady Aptek..

[51] American College of Clinical Pharmacy (2014). Standards of practice for clinical pharmacists. Pharmacotherapy.

[52] Garcia S.I., Rodrígez-González C.G., Martín-Barbero M.L., Tovar-Pozo M. (2016). CP-085 The Impact of Pharmacist Interventions on Safety and Cost Savings. Eur. J. Hosp. Pharm..

[53] Gallagher J., Byrne S., Woods N., Lynch D., McCarthy S. (2014). Cost-outcome description of clinical pharmacist interventions in a university teaching hospital. BMC Health Serv. Res..

[54] Khalili H., Karimzadeh I., Mirzabeigi P., Dashti-Khavidaki S. (2013). Evaluation of clinical pharmacist’s interventions in an infectious diseases ward and impact on patient’s direct medication cost. Eur. J. Intern. Med..

[55] Khalili H., Farsaei S., Rezaee H., Dashti-Khavidaki S. (2011). Role of clinical pharmacists’ interventions in detection and prevention of medication errors in a medical ward. Int. J. Clin. Pharm..

[56] Alderman C.P., Farmer C. (2001). A brief analysis of clinical pharmacy interventions undertaken in an Australian teaching hospital. J. Qual. Clin. Pract..

[57] Krämer I. Hospital Pharmacy in Germany. https://hospitalpharmacyeurope.com/news/editors-pick/hospital-pharmacy-in-germany.

[58] Ronan S., Shannon N., Cooke K., McKeon T., Walsh E.K., Kearney A., Sahm L.J. (2020). The Role of the Clinical Pharmacist in an Irish University Teaching Hospital: A Mixed-Methods Study. Pharmacy.

[59] NHS Cumbria Clinical Commissioning Group (2013). Medication Review. A Practice Guide. https://medicines.necsu.nhs.uk/cumbria-practice-resources.

[60] Corsonello A., Pedone C., Incalzi R.A. (2010). Age-related pharmacokinetic and pharmacodynamic changes and related risk of adverse drug reactions. Curr. Med. Chem..

[61] Beers M.H. (1999). Aging as a Risk Factor for Medication-Related Problems. Consult. Pharm..

[62] FIP (2021). Medicines Reconciliation. A Toolkit for Pharmacists. https://www.fip.org/file/4949.

[63] Kearney A., Halleran C., Walsh E., Byrne D., Haugh J., Sahm L.J. (2017). Medication Reviews by a Clinical Pharmacist at an Irish University Teaching Hospital. Pharmacy.

[64] Bem A. (2013). Public financing of healthcare services. Efinanse—Financ. Internet Q..

