# Supplementary material for: Directions of Changes in the Profession of Hospital Pharmacist in Poland
Source: Int J Environ Res Public Health. 2022 Nov 5;19(21):14522. doi: 10.3390/ijerph192114522 (PMC9658758; doi:10.3390/ijerph192114522)
Supplement: Supplementary file 1 [file ijerph-19-14522-s001.zip › Questionnaire_ Surveymonkey en-GB.pdf]

DeepL

Subscribe to DeepL Pro to edit this document.
Visit www.DeepL.com/profor more information.

Change in the hospital pharmacy profession

1. survey on the role of the hospital pharmacist

The research survey is being conducted as part of the diploma project 'Change in the model of functioning of the hospital pharmacy profession in the Polish healthcare system' as part of the postgraduate MBA in healthcare.

The questionnaire contains proposals that have been made as part of the work of the Team for the development of solutions for clinical pharmacy and the activities of pharmacists in medical entities, together with a list of diagnostic tests performed by pharmacists appointed by the Minister of Health.

Your feedback will allow me both to prepare my thesis and, above all, to propose optimal solutions that will shape our profession in the coming years.

I kindly ask you to fill in the questionnaire completely.
The survey contains 37 questions grouped into 8 pages. The estimated time required to complete the survey is 15 minutes.

Thank you very much in advance for your time and work.
pharm.mgr. Marcin Bochniarz

* 1. Please confirm that you hold a professional title of Master of Pharmacy and that you work in a hospital (treatment facility): 0

- YES
- NO (ends survey)

* 2. Please indicate your position/position in the pharmacy 0.

- Pharmacy manager - public sector
- Pharmacist
- Intern
- Owner or partner and pharmacy manager - private hospital sector
- Owner or partner of the pharmacy, but not the manager
- Pharmacy manager but not owner or partner - private hospital sector
- Other

* 3. Number of years in pharmacy (if not applicable please enter 0): 0

Please indicate your age:

Number of years working in pharmacy:

Number of years working in hospital pharmacy (public sector):

Number of years in hospital pharmacy (private sector):

Number of years in current position:

* 4. Sex 0

- Woman

Male

* 5. education: 0

MA pharmacy
dr n. pharm.
dr hab. n. farm.
prof. dr hab. n. farm.

* 6. Do you have a specialisation in pharmacy? 0

NO
yes - hospital pharmacy
yes - clinical pharmacy
yes - pharmacology
yes - pharmacy pharmacy
yes - other

* 7. please choose the province in which you work: 0

* 8. specify the size of the treatment facility where you work: 0

up to 100 beds
101 - 300 beds
301 - 600 beds
601 - 1000 beds
>1000 beds

NEW QUESTION

or Copy and paste questions

Further

Supported by
See how easy it is to create a survey.

0 of 37 with answers

NEW PAGE

P2: Need for change

Page Logic More Actions

UPGRADE TO ADD A LOGO

Change in the hospital pharmacy profession

2. the need for change

* 9. Please indicate to what extent you derive **satisfaction** from the way you currently practise as a pharmacist (0-10)? 0

0 (no satisfaction) 5 10 (complete satisfaction)

* 10. Do you think there needs to be a change in the model of functioning of pharmacists in medical entities? 0

- YES
- NO

* 11. Please rate on a scale of 1-10 your **overall willingness** to work on making changes to the way pharmacists function in your hospital: 0

0 (not ready) 5 10 (full readiness)

NEW QUESTION

or Copy and paste questions

Back Further

Supported by
See how easy it is to create a survey.

0 of 37 with answers

NEW PAGE

P3: Change in the distribution area

-

Page Logic More Actions

UPGRADE TO ADD A LOGO

Change in the hospital pharmacy profession

3. change in the distribution area

* 12. Do you think there needs to be a change in the way pharmacists operate in the **distribution of** medicinal products and medical devices? 0

- YES
- NO

* 13. Please rate on a scale of 1-5 the **necessity of the** following changes in the distribution area, where 1 means no necessity and 5 means absolute necessity: 0

	1 (not necessary)	2	3	4	5 (absolute necessity)
introduction of automation in the area of distribution of medicinal products in	Introduction of automation in the area of medicines	Introduction of automation in the area of medicinal	Introduction of automation in the area of distribution of	Introduction of automation in the area of distribution of	Introduction of automation in the area of pharmacy

	1 (not necessary)	2	3	4	5 (absolute necessity)
the pharmacy (robots receiving, storing and dispensing medicines in full packs)	distribution in the pharmacy (robots receiving, storing and dispensing medicines in full packs) 1 (not necessary)	product distribution in the pharmacy (robots receiving, storing and dispensing medicines in full packs) 2	medicinal products in the pharmacy (robots receiving, storing and dispensing medicines in full packs) 3	medicinal products in the pharmacy (robots receiving, storing and dispensing medicines in full packs) 4	distribution of medicines (robots receiving, storing and dispensing medicines in full packs) 5 (absolute necessity)
the introduction of a unit-dose system, i.e. a system for preparing individual doses of ready-made medicines in the pharmacy for an individual patient	introduction of a unit-dose system, i.e. a system for preparing individual doses of ready-to-use medicines in the pharmacy for an individual patient 1 (not necessary)	introduction of a unit-dose system, i.e. a system for preparing individual doses of ready-to-use medicines in the pharmacy for an individual patient 2	introduction of a unit-dose system, i.e. a system for preparing individual doses of ready-to-use medicines in the pharmacy for an individual patient 3	introduction of a unit-dose system, i.e. a system for preparing individual doses of ready-to-use medicines in the pharmacy for an individual patient 4	introduction of a unit-dose system, i.e. a system for preparing individual doses of ready-made medicines in the pharmacy for an individual patient 5 (an absolute necessity)
introduction of an automatic filing cabinet system in hospital wards	introduction of a system of automatic medicine cabinets in hospital wards 1 (not necessary)	introduction of an automatic filing cabinet system in hospital wards 2	introduction of an automatic filing cabinet system in hospital wards 3	introduction of an automatic filing cabinet system in hospital wards 4	introduction of a system of automatic medicine cabinets in hospital wards 5 (an absolute necessity)
introduction of a system that tracks the medication in the hospital from order, through preparation for administration, to administration to the patient (e.g. a drug coding system that verifies and remembers the medication given to an individual patient)	introduction of a system that tracks the medication in the hospital from order, through preparation for administration, to administration to the patient (e.g. a medication coding system that verifies and remembers the medication given to an individual patient) 1 (not necessary)	introduction of a system that tracks the medication in the hospital from order, through preparation for administration, to administration to the patient (e.g. a medication coding system that verifies and remembers the medication given to an individual patient) 2	introduction of a system that tracks the medication in the hospital from order, through preparation for administration, to administration to the patient (e.g. a medication coding system that verifies and remembers the medication given to an individual patient) 3	introduction of a system that tracks the medication in the hospital from order, through preparation for administration, to administration to the patient (e.g. a medication coding system that verifies and remembers the medication given to an individual patient) 4	introduction of a system that tracks the medication in the hospital from order, through preparation for administration, to administration to the patient (e.g. a drug coding system that verifies and remembers the medication given to an individual patient) 5 (absolute necessity)
digitisation of pharmacotherapy processes in the hospital (electronic medical order sheets, electronic requisitions to the hospital pharmacy, electronic orders, etc.).	Digitisation of pharmacotherapy processes in the hospital (electronic medical order sheets, electronic requisitions to the hospital pharmacy, electronic orders, etc.) 1 (not necessary)	digitisation of pharmacotherapy processes in the hospital (electronic medical order sheets, electronic requisitions to the hospital pharmacy, electronic orders, etc.) 2	digitisation of pharmacotherapy processes in the hospital (electronic medical order sheets, electronic requisitions to the hospital pharmacy, electronic orders, etc.). 3	digitisation of pharmacotherapy processes in the hospital (electronic medical order sheets, electronic requisitions to the hospital pharmacy, electronic orders, etc.). 4	digitisation of pharmacotherapy processes in the hospital (electronic medical order sheets, electronic requisitions to the hospital pharmacy, electronic orders, etc.) 5 (absolute necessity)
educational programmes in the area of risk assessment and quality management for hospital pharmacists (e.g. process management, lean management solutions)	educational programmes in the area of risk assessment and quality management for hospital pharmacists (e.g. process management, lean management solutions) 1 (not necessary)	educational programmes in the area of risk assessment and quality management for hospital pharmacists (e.g. process management, lean management solutions) 2	educational programmes in the area of risk assessment and quality management for hospital pharmacists (e.g. process management, lean management solutions) 3	educational programmes in the area of risk assessment and quality management for hospital pharmacists (e.g. process management, lean management solutions) 4	educational programmes in the area of risk assessment and quality management for hospital pharmacists (e.g. process management, lean management solutions) 5 (absolute necessity)
development and introduction of nationwide quality standards in hospital pharmacy, regulating the principles of conduct in individual departments	Development and introduction of nationwide quality standards in hospital pharmacy, regulating the rules of conduct in individual departments 1 (not necessary)	development and introduction of nationwide quality standards in hospital pharmacy, regulating the principles of conduct in individual departments 2	development and introduction of nationwide quality standards in hospital pharmacy, regulating the principles of conduct in individual departments 3	development and introduction of nationwide quality standards in hospital pharmacy, regulating the principles of conduct in individual departments 4	Development and introduction of nationwide quality standards in hospital pharmacy, regulating the principles of conduct in individual departments 5 (an absolute necessity)

	1 (not necessary)	2	3	4	5 (absolute necessity)
developing new requirements for the premises of hospital pharmacies and hospital pharmacy departments, taking into account current developments in pharmacy	development of new requirements for the premises of the hospital pharmacy and the hospital pharmacy department, taking into account current developments in pharmacy 1 (not necessary)	to develop new requirements for the premises of the hospital pharmacy and the hospital pharmacy department, taking into account current developments in pharmacy 2	to develop new requirements for the premises of the hospital pharmacy and the hospital pharmacy department, taking into account current developments in pharmacy 3	to develop new requirements for the premises of the hospital pharmacy and the hospital pharmacy department, taking into account current developments in pharmacy 4	to develop new requirements for the premises of the hospital pharmacy and the hospital pharmacy department, taking into account current developments in pharmacy 5 (an absolute necessity)

14. If you think other changes in the distribution area are needed, please write them down: 0

* Please rate on a scale of 1-10 your **readiness** to work on the introduction of new solutions in the distribution area, where 1 indicates no readiness and 10 indicates full readiness. 0

0 (not ready) 5 10 (full readiness)

NEW QUESTION

or Copy and paste questions

Back Further

Supported by
See how easy it is to create a survey.

0 of 37 with answers

NEW PAGE

P4: Change in the area of drafting

Page Logic More Actions

UPGRADE TO ADD A LOGO

Change in the hospital pharmacy profession

4. change in the area of drafting

* 16. Do you think changes are needed in the way pharmacists operate in the area of **preparing medicinal products**? 0

- YES
- NO

* 17. Please rate on a scale of 1-5 the **necessity of the** following changes in the area of medicinal product preparation, where 1 means no necessity and 5 means absolute necessity:

0

	1 (not necessary)	2	3	4	5 (absolute necessity)
Introducing the preparation risk assessment	Introduce the preparation risk assessment	Introduce the preparation risk assessment	Introduce the preparation risk assessment	Introduce the preparation risk assessment	Introduce the preparation risk assessment

	1 (not necessary)	2	3	4	5 (absolute necessity)
methodology as a tool that decides on the form of drug purchased by the hospital (RTA, RTU, drug requiring preparation) and helps to indicate where the drug should be prepared for administration:	methodology as a tool that decides on the form of drug purchased by the hospital (RTA, RTU, drug requiring preparation) and helps to indicate where the drug should be prepared for administration: 1 (not necessary)	methodology as a tool that decides on the form of drug purchased by the hospital (RTA, RTU, drug requiring preparation) and helps to indicate where the drug should be prepared for administration: 2	methodology as a tool that decides on the form of drug purchased by the hospital (RTA, RTU, drug requiring preparation) and helps to indicate where the drug should be prepared for administration: 3	methodology as a tool that decides on the form of drug purchased by the hospital (RTA, RTU, drug requiring preparation) and helps to indicate where the drug should be prepared for administration: 4	methodology as a tool that decides on the form of drug purchased by the hospital (RTA, RTU, drug requiring preparation) and helps to indicate where the drug should be prepared for administration: 5 (absolute necessity)
the introduction of a taxa laborum fee for the preparation of sterile medicinal products in hospital pharmacies:	Introduction of a taxa laborum fee for the preparation of sterile medicinal products in hospital pharmacies: 1 (not necessary)	the introduction of a taxa laborum for the preparation of sterile medicinal products in hospital pharmacies: 2	introduction of a taxa laborum for the preparation of sterile medicinal products in hospital pharmacies: 3	introduction of a taxa laborum for the preparation of sterile medicinal products in hospital pharmacies: 4	Introduction of a taxa laborum for the preparation of sterile medicinal products in hospital pharmacies: 5 (absolute necessity)
Allowing excess directly funded drugs (cytostatics, drugs in drug programmes) to be billed to the NHF:	Allowing excess directly funded drugs (cytostatics, drugs in drug programmes) to be billed to the NHF: 1 (no need)	Allowing excess directly funded drugs (cytostatics, drugs in drug programmes) to be billed to the NHF: 2	Allowing excess directly funded drugs (cytostatics, drugs in drug programmes) to be billed to the NHF: 3	Allowing excess directly funded drugs (cytostatics, drugs in drug programmes) to be billed to the NHF: 4	Allowing excess directly funded drugs (cytostatics, drugs in drug programmes) to be billed to the NHF: 5 (absolute necessity)

18. if you think other changes are needed in the drafting area, please write them in: 0

* Please rate on a scale of 1-10 your **willingness** to introduce new developments in the area of medicinal product preparation: [1-10] 0

0 (not ready) 5 10 (full readiness)

NEW QUESTION

or Copy and paste questions

Back Further

Supported by
See how easy it is to create a survey.

0 of 37 with answers

NEW PAGE

P5: Research and development

-

Page Logic More Actions

UPGRADE TO ADD A LOGO

Change in the hospital pharmacy profession

5. research and development

* 20. Do you think that **research and development (R&D) work should be carried out** in Polish hospital pharmacies**,** e.g. in cooperation with a university or a private company? Examples of areas include drug form technology or new drug technologies. 0

- YES
- NO

* 21. Please rate on a scale of 1-5 the **necessity of** the following changes in the area of research and development (R&D), where 1 means no necessity and 5 means absolute necessity: 0

	1 (not necessary)	2	3	4	5 (absolute necessity)
preparation of innovative medicinal products in hospital pharmacies, e.g. advanced therapy medicinal products (e.g. gene therapy, CAR-T therapy):	Preparation of innovative medicinal products in hospital pharmacies, e.g. advanced therapy medicinal products (e.g. gene therapy, CAR-T therapy): 1 (not necessary)	preparation of innovative medicinal products in hospital pharmacies, e.g. advanced therapy medicinal products (e.g. gene therapy, CAR-T therapy): 2	preparation of innovative medicinal products in hospital pharmacies, e.g. advanced therapy medicinal products (e.g. gene therapy, CAR-T therapy): 3	preparation of innovative medicinal products in hospital pharmacies, e.g. advanced therapy medicinal products (e.g. gene therapy, CAR-T therapy): 4	preparation of innovative medicinal products in hospital pharmacies, e.g. advanced therapy medicinal products (e.g. gene therapy, CAR-T therapy): 5 (absolute necessity)
research in the area of innovative drug formulation and dose individualisation (e.g. 3D printing of drugs):	research in the area of innovative drug formulation and dose individualisation (e.g. 3D printing of drugs): 1 (no need)	research in the area of innovative drug formulation and dose individualisation (e.g. 3D printing of drugs): 2	research in the area of innovative drug formulation and dose individualisation (e.g. 3D printing of drugs): 3	research in the area of innovative drug formulation and dose individualisation (e.g. 3D printing of drugs): 4	research in the area of innovative drug formulation and dose individualisation (e.g. 3D printing of drugs): 5 (absolute necessity)
R&D cooperation with universities in areas relating to pharmacotherapy:	R&D cooperation with universities in areas related to pharmacotherapy: 1 (not necessary)	cooperation with universities on R&D in areas relating to pharmacotherapy: 2	cooperation with universities on R&D in areas relating to pharmacotherapy: 3	cooperation with universities on R&D in areas relating to pharmacotherapy: 4	R&D cooperation with universities in areas related to pharmacotherapy: 5 (absolute necessity)
cooperation with private companies for R&D in areas concerning pharmacotherapy:	Collaboration with private companies for R&D in areas related to pharmacotherapy: 1 (not necessary)	cooperation with private companies for R&D in areas concerning pharmacotherapy: 2	cooperation with private companies for R&D in areas concerning pharmacotherapy: 3	cooperation with private companies for R&D in areas concerning pharmacotherapy: 4	Collaboration with private companies for R&D in areas related to pharmacotherapy: 5 (absolute necessity)
participation in clinical trials of medicinal products	participation in clinical trials on medicinal products 1 (not necessary)	participation in clinical trials on medicinal products 2	participation in clinical trials of medicinal products 3	participation in clinical trials of medicinal products 4	participation in clinical trials of medicinal products 5 (absolute necessity)
participation in clinical trials of medical devices	participation in clinical trials of medical devices 1 (not necessary)	participation in clinical trials of medical devices 2	participation in clinical trials of medical devices 3	participation in clinical trials of medical devices 4	participation in clinical trials of medical devices 5 (absolute necessity)

22. if you think other changes are needed in the field of R&D, please write them in: 0

* 23. Please rate on a scale of 1-10 your **readiness** to introduce new solutions in the field of research and development: 0

0 (not ready) 5 10 (full readiness)

NEW QUESTION

or Copy and paste questions

Back Further

Supported by
See how easy it is to create a survey.

NEW PAGE

P6: Clinical pharmacy - general section

Page Logic More Actions

UPGRADE TO ADD A LOGO

Change in the hospital pharmacy profession

6. clinical pharmacy - general section

* 24. Do you think there needs to be a change in the way pharmacists function in the hospital by **setting up clinical pharmacy services?** 0

- YES
- NO

* 25. Please rate on a scale of 1-10 your overall **readiness** to work on setting up clinical pharmacy services in your hospital: 0

0 (not ready) 5 10 (full readiness)

* 26. In your opinion, do you have the necessary **knowledge** to start providing clinical pharmacy services? 0

- YES
- NO

* 27. In your opinion, do you currently have the necessary **skills** to start providing clinical pharmacy services? 0

- YES
- NO

* 28. In your opinion, do you currently have the **tools (e.g. procedure, manual, patient questionnaire)** necessary to start providing clinical pharmacy services? 0

- YES
- NO

* 29. Do you have the necessary **time** at work to start and run clinical pharmacy services at your workplace? 0

- YES
- NO

* 30. Where do you think the clinical pharmacist should be employed: 0

- in a hospital pharmacy (reporting to the head of pharmacy)
- in a hospital pharmacy provided that a team of clinical pharmacists is separated and work is provided exclusively in this area (reporting to the pharmacy manager)
- directly in the department (reporting to the head of department)
- in a separate clinical pharmacy team that provides services to the whole hospital (the clinical pharmacist reports to the pharmacist who is the head of this team).

NEW QUESTION

or Copy and paste questions

Back Further

Supported by
See how easy it is to create a survey.

NEW PAGE

P7: Clinical pharmacy - services

Page Logic More Actions

UPGRADE TO ADD A LOGO

Change in the hospital pharmacy profession

7. clinical pharmacy - services

* Please rate your **knowledge of** each clinical pharmacy tool, where 1 means no knowledge and 5 means full knowledge of the tool: 0

	1 (no knowledge)	2	3	4	5 (full knowledge)
Drug conciliation	Drug conciliation 1 (no knowledge)	Drug conciliation 2	Drug conciliation 3	Drug conciliation 4	Drug conciliation 5 (full knowledge)
Drug review	Drug review 1 (no knowledge)	Drug review 2	Drug review 3	Drug review 4	Drug review 5 (full knowledge)
Development of an individual pharmaceutical care plan	Development of an individual pharmaceutical care plan 1 (no knowledge)	Development of an individual pharmaceutical care plan 2	Development of an individual pharmaceutical care plan 3	Development of an individual pharmaceutical care plan 4	Development of an individual pharmaceutical care plan 5 (full knowledge)
Education in the field of pharmacotherapy directed at patients or medical staff	Education in the field of pharmacotherapy directed at patients or medical staff 1 (not known)	Education in the field of pharmacotherapy directed at patients or medical staff 2	Education in the field of pharmacotherapy directed at patients or medical staff 3	Education in the field of pharmacotherapy directed at patients or medical staff 4	Education in the field of pharmacotherapy directed at patients or medical staff 5 (full knowledge)
Conducting drug concentration-monitored therapy	Provision of therapy monitored by blood drug concentration 1 (not known)	Conducting drug concentration-monitored therapy 2	Conducting drug concentration-monitored therapy 3	Conducting drug concentration-monitored therapy 4	Conducting drug concentration-monitored therapy 5 (full knowledge)

	1 (no knowledge)	2	3	4	5 (full knowledge)
Individual consultation on the patient's pharmacotherapy as ordered by the doctor	Individual consultation on the patient's pharmacotherapy as ordered by the doctor 1 (no knowledge)	Individual consultation on the patient's pharmacotherapy as ordered by the doctor 2	Individual consultation on the patient's pharmacotherapy as ordered by the doctor 3	Individual consultation on the patient's pharmacotherapy as ordered by the doctor 4	Individual consultation on the patient's pharmacotherapy as ordered by the doctor 5 (full knowledge)
Creation of a hospital antibiotic policy	Creation of hospital antibiotic policy 1 (lack of knowledge)	Creation of a hospital antibiotic policy 2	Creation of a hospital antibiotic policy 3	Creation of a hospital antibiotic policy 4	Creation of hospital antibiotic policy 5 (full knowledge)
Consulting the patient's individual antibiotic therapy	Consulting a patient's individual antibiotic therapy 1 (no knowledge)	Consulting a patient's individual antibiotic therapy 2	Consulting a patient's individual antibiotic therapy 3	Consulting the patient's individual antibiotic therapy 4	Consulting a patient's individual antibiotic therapy 5 (full knowledge)
Participation in ward rounds and interdisciplinary consiliums	Participation in ward rounds and interdisciplinary consiliums 1 (no knowledge)	Participation in ward rounds and interdisciplinary consiliums 2	Participation in ward rounds and interdisciplinary consiliums 3	Participation in ward rounds and interdisciplinary consiliums 4	Participation in ward rounds and interdisciplinary consiliums 5 (full knowledge)
Issuing follow-up prescriptions, e.g. for the administration of a further cycle of chemotherapy to a patient (according to the regimen ordered by the doctor):	Issue continuation prescriptions, e.g. for the administration of a further cycle of chemotherapy to a patient (according to the regimen ordered by the doctor): 1 (no knowledge)	Issue continuation prescriptions, e.g. for the administration of a further cycle of chemotherapy to a patient (according to the regimen ordered by the doctor): 2	Issue continuation prescriptions, e.g. for the administration of a further cycle of chemotherapy to a patient (according to the regimen ordered by the doctor): 3	Issue continuation prescriptions, e.g. for the administration of a further cycle of chemotherapy to a patient (according to the regimen ordered by the doctor): 4	Issue continuation prescriptions, e.g. for the administration of a further cycle of chemotherapy to a patient (as prescribed by the doctor): 5 (full knowledge)

* 32. Please rate on a scale of 1-5 the **need for the** following clinical pharmacy services, where 1 means no need and 5 means an absolute need for the tool: 0

	1 (not necessary)	2	3	4	5 (absolute necessity)
drug conciliation	drug conciliation 1 (not necessary)	drug conciliation 2	drug conciliation 3	drug conciliation 4	drug conciliation 5 (absolute necessity)
drug review	drug review 1 (not necessary)	drug review 2	drug review 3	drug review 4	drug review 5 (absolute necessity)
development of an individual pharmaceutical care plan	development of an individual pharmaceutical care plan 1 (not necessary)	development of an individual pharmaceutical care plan 2	development of an individual pharmaceutical care plan 3	development of an individual pharmaceutical care plan 4	development of an individual pharmaceutical care plan 5 (absolute necessity)
education in the field of pharmacotherapy directed at patients or medical staff	Pharmacovigilance education for patients or medical staff 1 (not necessary)	education in the field of pharmacotherapy directed at patients or medical staff 2	pharmacovigilance education for patients or medical staff 3	pharmacovigilance education for patients or medical staff 4	pharmacovigilance education for patients or healthcare staff 5 (absolute necessity)
conducting blood-concentration-monitored therapy	conducting blood-concentration-monitored therapy 1 (not necessary)	conducting blood-concentration-monitored therapy 2	conducting blood-concentration-monitored therapy 3	conduct of blood-concentration-monitored therapy 4	conduct of blood-concentration-monitored therapy 5 (absolute necessity)
individual consultation on the patient's	individual consultation on the patient's	individual consultation on the patient's	individual consultation on the patient's	individual consultation on the patient's	individual consultation on the patient's

	1 (not necessary)	2	3	4	5 (absolute necessity)
pharmacotherapy as ordered by the doctor	pharmacotherapy as ordered by the doctor 1 (not necessary)	pharmacotherapy as ordered by the doctor 2	pharmacotherapy as ordered by the doctor 3	pharmacotherapy as ordered by the doctor 4	pharmacotherapy as ordered by the doctor 5 (absolute necessity)
the creation of a hospital antibiotics policy	creation of a hospital antibiotic policy 1 (not necessary)	creating a hospital antibiotic policy 2	the development of a hospital antibiotic policy 3	development of hospital antibiotic policy 4	creation of a hospital antibiotic policy 5 (an absolute necessity)
consulting the patient's individual antibiotic therapy	consulting an individual patient's antibiotic therapy 1 (not necessary)	consulting the patient's individual antibiotic therapy 2	consulting the patient's individual antibiotic therapy 3	consulting the patient's individual antibiotic therapy 4	consulting the patient's individual antibiotic therapy 5 (absolute necessity)
participation in ward rounds and interdisciplinary consultations	Participation in ward rounds and interdisciplinary consiliums 1 (not necessary)	participation in ward rounds and interdisciplinary consiliums 2	participation in ward rounds and interdisciplinary consiliums 3	participation in ward rounds and interdisciplinary consiliums 4	participation in ward rounds and interdisciplinary consiliums 5 (absolute necessity)
issuing follow-up prescriptions, e.g. for the administration of the next cycle of chemotherapy to the patient (according to the regimen ordered by the doctor):	issuing follow-up prescriptions, e.g. for the administration of a further cycle of chemotherapy to a patient (according to the regimen ordered by the doctor): 1 (not necessary)	issuing follow-up prescriptions, e.g. for the administration of a further cycle of chemotherapy to a patient (according to the regimen ordered by the doctor): 2	issuing follow-up prescriptions, e.g. for the administration of a further cycle of chemotherapy to a patient (according to the regimen ordered by the doctor): 3	issuing follow-up prescriptions, e.g. for the administration of a further cycle of chemotherapy to a patient (according to the regimen ordered by the doctor): 4	issuing continuation prescriptions, e.g. for the administration of the next cycle of chemotherapy to the patient (according to the regimen ordered by the doctor): 5 (absolute necessity)

33 Please rate on a scale of 1-5 your **readiness** to introduce the following clinical pharmacy services in your hospital, , where 1 indicates no readiness and 5 indicates full readiness to implement the change: 0

	1 (not ready)	2	3	4	5 (full readiness)
drug conciliation	drug conciliation 1 (unpreparedness)	drug conciliation 2	drug conciliation 3	drug conciliation 4	drug conciliation 5 (full readiness)
drug review	drug review 1 (not ready)	drug review 2	drug review 3	drug review 4	drug review 5 (full readiness)
development of an individual pharmaceutical care plan	development of an individual pharmaceutical care plan 1 (not ready)	development of an individual pharmaceutical care plan 2	development of an individual pharmaceutical care plan 3	development of an individual pharmaceutical care plan 4	development of an individual pharmaceutical care plan 5 (full readiness)
education in the field of pharmacotherapy directed at patients or medical staff	Education in the field of pharmacotherapy directed at patients or medical staff 1 (not ready)	education in the field of pharmacotherapy directed at patients or medical staff 2	pharmacovigilance education for patients or healthcare professionals 3	pharmacovigilance education for patients or medical staff 4	Pharmacovigilance education for patients or medical staff 5 (full readiness)
conducting blood-concentration-monitored therapy	conducting drug concentration-monitored therapy 1 (not ready)	conducting blood-concentration-monitored therapy 2	conducting blood-concentration-monitored therapy 3	conduct of blood-concentration-monitored therapy 4	conduct of blood-concentration-monitored therapy 5 (full readiness)

	1 (not ready)	2	3	4	5 (full readiness)
individual consultation on the patient's pharmacotherapy as ordered by the doctor	individual consultation on the patient's pharmacotherapy as ordered by the doctor 1 (not ready)	individual consultation on the patient's pharmacotherapy as ordered by the doctor 2	individual consultation on the patient's pharmacotherapy as ordered by the doctor 3	individual consultation on the patient's pharmacotherapy as ordered by the doctor 4	individual consultation on the patient's pharmacotherapy as ordered by the doctor 5 (full readiness)
the creation of a hospital antibiotics policy	creation of a hospital antibiotic policy 1 (lack of preparedness)	creating a hospital antibiotic policy 2	the development of a hospital antibiotic policy 3	development of hospital antibiotic policy 4	creation of a hospital antibiotic policy 5 (full readiness)
consulting the patient's individual antibiotic therapy	consulting individual antibiotic therapy of patient 1 (not ready)	consulting the patient's individual antibiotic therapy 2	consulting the patient's individual antibiotic therapy 3	consulting the patient's individual antibiotic therapy 4	consulting an individual patient's antibiotic therapy 5 (full readiness)
participation in ward rounds and interdisciplinary consultations	Participation in ward rounds and interdisciplinary consiliums 1 (not ready)	participation in ward rounds and interdisciplinary consiliums 2	participation in ward rounds and interdisciplinary consiliums 3	participation in ward rounds and interdisciplinary consiliums 4	Participation in ward rounds and interdisciplinary consiliums 5 (full time)
issuing follow-up prescriptions, e.g. for the administration of the next cycle of chemotherapy to the patient (according to the regimen ordered by the doctor):	issuing follow-up prescriptions, e.g. for the administration of a further cycle of chemotherapy to a patient (according to the regimen ordered by the doctor): 1 (not ready)	issuing follow-up prescriptions, e.g. for the administration of a further cycle of chemotherapy to a patient (according to the regimen ordered by the doctor): 2	issuing follow-up prescriptions, e.g. for the administration of a further cycle of chemotherapy to a patient (according to the regimen ordered by the doctor): 3	issuing follow-up prescriptions, e.g. for the administration of a further cycle of chemotherapy to a patient (according to the regimen ordered by the doctor): 4	issuing follow-up prescriptions, e.g. for the administration of a further cycle of chemotherapy to a patient (according to the regimen ordered by the doctor): 5 (full readiness)

34. If you think that other clinical pharmacy services are also worth introducing, please name them: 0

NEW QUESTION

or Copy and paste questions

Back Further

Supported by
See how easy it is to create a survey.

0 of 37 with answers

NEW PAGE

P8: Clinical pharmacy - completion

Page Logic More Actions

UPGRADE TO ADD A LOGO

Change in the hospital pharmacy profession

8 Clinical pharmacy - completion

* 35. Are you concerned about the increased workload associated with the launch of new clinical activities for pharmacists? 0

- YES
- NO

0 UPGRADE TO EDIT OPTIONS LOGIC MOVE COPY LIBRARY DELETE

* 36. Please rate the possible **obstacles** to the introduction of clinical pharmacy services in Polish hospitals, where 1 means that it is not an obstacle and 5 that it is a barrier, without overcoming which it will not be possible to implement the change: 0

	1 (this is not an obstacle)	2	3	4	5 (this is an obstacle blocking change)
lack of sufficient professional staff	lack of sufficient professional staff 1 (this is not an obstacle)	lack of sufficient professional staff 2	lack of sufficient professional staff 3	lack of sufficient professional staff 4	lack of sufficient professional staff 5 (this is an obstacle blocking change)
lack of knowledge to provide such services by pharmacists	Lack of knowledge to provide this type of service by pharmacists 1 (this is not an obstacle)	lack of knowledge to provide this type of service by pharmacists 2	lack of knowledge to provide this type of service by pharmacists 3	lack of knowledge to provide this type of service by pharmacists 4	Lack of knowledge to provide this type of service by pharmacists 5 (this is an obstacle blocking change)
the lack of skills of pharmacists to provide this type of service	Lack of skills to provide this type of service by pharmacists 1 (this is not an obstacle)	Lack of capacity to provide such services by pharmacists 2	Lack of skills of pharmacists to provide this type of service 3	Lack of capacity to provide such services by pharmacists 4	Lack of skills to provide this type of service by pharmacists 5 (this is an obstacle blocking change)
lack of payer funding for clinical pharmacy services	lack of payer funding for clinical pharmacy services 1 (this is not an obstacle)	lack of payer funding for clinical pharmacy services 2	lack of payer funding for clinical pharmacy services 3	lack of payer funding for clinical pharmacy services 4	lack of payer funding for clinical pharmacy services 5 (this is an obstacle blocking change)

37 Please list any other barriers, if any, to the introduction of clinical pharmacy services: 0

NEW QUESTION
