# Supplementary material for: Directions of Changes in the Profession of Hospital Pharmacist in Poland
Source: Int J Environ Res Public Health. 2022 Nov 5;19(21):14522. doi: 10.3390/ijerph192114522 (PMC9658758; doi:10.3390/ijerph192114522)
Supplement: Supplementary file 1 [file ijerph-19-14522-s001.zip › Questionnaire_ Surveymonkey.pdf]

Zmiana w zawodzie farmaceuty szpitalnego

1. Ankieta dotycząca roli farmaceuty szpitalnego

Ankieta badawcza przeprowadzana jest w ramach projektu dyplomowego „Zmiana w modelu funkcjonowania zawodu farmaceuty szpitalnego w polskim systemie ochrony zdrowia" w ramach studiów podyplomowych MBA w ochronie zdrowia.

Ankieta zawiera propozycje, które zostały zgłoszone w ramach prac Zespołu do spraw wypracowania rozwiązań w zakresie farmacji klinicznej oraz działalności farmaceutów w podmiotach leczniczych wraz wykazem badań diagnostycznych wykonywanych przez farmaceutów powołanego przez Ministra Zdrowia.

Państwa opinia pozwoli mi zarówno na przygotowanie pracy dyplomowej, jak i przede wszystkim na zaproponowanie optymalnych rozwiązań, które będą w najbliższych latach kształtowały nasz zawód.

Uprzejmie proszę o kompletne wypełnienie ankiety.
Ankieta zawiera 37 pytań pogrupowanych na 8 stronach. Szacowany czas niezbędny do wypełnienia ankiety to 15 minut.

Z góry bardzo dziękuję za Państwa czas i pracę.
mgr farm. Marcin Bochniarz

* 1. Proszę potwierdzić, że posiada Pani/Pan tytuł zawodowy magistra farmacji i pracuje w szpitalu (podmiocie leczniczym): 0

- TAK
- NIE (kończy ankietę)

* 2. Proszę podać swoją pozycję/stanowisko w aptece 0

- Kierownik apteki - sektor publiczny
- Farmaceuta
- Stażysta
- Właściciel lub wspólnik i kierownik apteki - sektor szpitali prywatnych
- Właściciel lub partner apteki, ale nie kierownik
- Kierownik apteki, ale nie właściciel ani partner - sektor szpitali prywatnych
- Inny

* 3. Liczba lat w farmacji (jeżeli nie dotyczy proszę wpisać 0): 0

Proszę podać swój wiek:

Liczba lat pracy w aptece:

Liczba lat pracy w aptece szpitalnej (sektor publiczny):

Liczba lat w aptece szpitalnej (sektor prywatny):

Liczba lat na obecnym stanowisku:

* 4. Płeć 0

- Kobieta
- Mężczyzna

* 5. Wykształcenie: 0

- mgr farmacji
- dr n. farm.
- dr hab. n. farm.
- prof. dr hab. n. farm.

* 6. Czy posiada Pani/Pan specjalizację w dziedzinie farmacji? 0

- NIE
- tak – farmacja szpitalna
- tak – farmacja kliniczna
- tak – farmakologia
- tak – farmacja apteczna
- tak – inna

* 7. Proszę wybrać województwo w którym Pani/Pan pracuje: 0

* 8. Proszę określić wielkość podmiotu leczniczego w którym Pani/Pan pracuje: 0

- do 100 łóżek
- 101 – 300 łóżek
- 301 – 600 łóżek
- 601 – 1000 łóżek
- >1000 łóżek

NEW QUESTION

or Copy and paste questions

Dalej

Obsługiwane przez
Zobacz, jak łatwo jest utworzyć ankietę.

0 z 37 z odpowiedziami

NEW PAGE

P2: Potrzeba zmiany

-

Page Logic | More Actions

UPGRADE TO ADD A LOGO

Zmiana w zawodzie farmaceuty szpitalnego

2. Potrzeba zmiany

* 9. Proszę o określenie w jakim stopniu czerpie Pani/Pan **satysfakcję** z aktualnego sposobu wykonywania przez Panią/Pana zawodu farmaceuty (0-10)? 0

0 (brak satysfakcji) 5 10 (pełna satysfakcja)

* 10. Czy uważa Pani/Pan, że konieczne jest wprowadzenie zmiany w modelu funkcjonowania farmaceutów w podmiotach leczniczych? 0

- TAK
- NIE

* 11. Proszę o ocenę w skali 1-10 Pani/Pana **ogólnej gotowości** do podjęcia pracy nad wprowadzeniem zmian w sposobie funkcjonowania farmaceutów w Państwa szpitalu: 0

0 (brak gotowości) 5 10 (pełna gotowość)

NEW QUESTION

or Copy and paste questions

Wstecz Dalej

Obsługiwane przez
Zobacz, jak łatwo jest utworzyć ankietę.

0 z 37 z odpowiedziami

NEW PAGE

P3: Zmiana w obszarze dystrybucyjnym

-

Page Logic More Actions

UPGRADE TO ADD A LOGO

Zmiana w zawodzie farmaceuty szpitalnego

3. Zmiana w obszarze dystrybucyjnym

* 12. Czy uważa Pani/Pan, że konieczne jest wprowadzenie zmiany w sposobie funkcjonowania farmaceutów w obszarze **dystrybucji** produktów leczniczych i wyrobów medycznych? 0

- TAK
- NIE

* 13. Proszę o ocenę w skali 1-5 **konieczności** wprowadzenia następujących zmian w obszarze dystrybucyjnym, gdzie 1 oznacza brak konieczności a 5 bezwzględną konieczność wprowadzenia zmiany: 0

	1 (brak konieczności)	2	3	4	5 (bezwzględna konieczność)
wprowadzenie automatyzacji w obszarze dystrybucji produktów leczniczych w aptece	wprowadzenie automatyzacji w obszarze dystrybucji produktów leczniczych w aptece	wprowadzenie automatyzacji w obszarze dystrybucji produktów leczniczych w aptece	wprowadzenie automatyzacji w obszarze dystrybucji produktów leczniczych w aptece	wprowadzenie automatyzacji w obszarze dystrybucji produktów leczniczych w aptece	wprowadzenie automatyzacji w obszarze dystrybucji produktów leczniczych w aptece

	1 (brak konieczności)	2	3	4	5 (bezwzględna konieczność)
(roboty przyjmujące, przechowujące i wydające leki w pełnych opakowaniach)	(roboty przyjmujące, przechowujące i wydające leki w pełnych opakowaniach) 1 (brak konieczności)	(roboty przyjmujące, przechowujące i wydające leki w pełnych opakowaniach) 2	(roboty przyjmujące, przechowujące i wydające leki w pełnych opakowaniach) 3	(roboty przyjmujące, przechowujące i wydające leki w pełnych opakowaniach) 4	(roboty przyjmujące, przechowujące i wydające leki w pełnych opakowaniach) 5 (bezwzględna konieczność)
wprowadzenie systemu unit-dose, tj. systemu przygotowywania indywidualnych dawek leków gotowych w aptece, z przeznaczeniem dla indywidualnego pacjenta	wprowadzenie systemu unit-dose, tj. systemu przygotowywania indywidualnych dawek leków gotowych w aptece, z przeznaczeniem dla indywidualnego pacjenta 1 (brak konieczności)	wprowadzenie systemu unit-dose, tj. systemu przygotowywania indywidualnych dawek leków gotowych w aptece, z przeznaczeniem dla indywidualnego pacjenta 2	wprowadzenie systemu unit-dose, tj. systemu przygotowywania indywidualnych dawek leków gotowych w aptece, z przeznaczeniem dla indywidualnego pacjenta 3	wprowadzenie systemu unit-dose, tj. systemu przygotowywania indywidualnych dawek leków gotowych w aptece, z przeznaczeniem dla indywidualnego pacjenta 4	wprowadzenie systemu unit-dose, tj. systemu przygotowywania indywidualnych dawek leków gotowych w aptece, z przeznaczeniem dla indywidualnego pacjenta 5 (bezwzględna konieczność)
wprowadzenie systemu automatycznych szaf lekowych na oddziałach szpitalnych	wprowadzenie systemu automatycznych szaf lekowych na oddziałach szpitalnych 1 (brak konieczności)	wprowadzenie systemu automatycznych szaf lekowych na oddziałach szpitalnych 2	wprowadzenie systemu automatycznych szaf lekowych na oddziałach szpitalnych 3	wprowadzenie systemu automatycznych szaf lekowych na oddziałach szpitalnych 4	wprowadzenie systemu automatycznych szaf lekowych na oddziałach szpitalnych 5 (bezwzględna konieczność)
wprowadzenie systemu śledzącego lek w szpitalu od zlecenia, poprzez przygotowanie do podania aż do podania pacjenta (np. system kodowania leków, który weryfikuje i zapamiętuje lek podany indywidualnemu pacjentowi)	wprowadzenie systemu śledzącego lek w szpitalu od zlecenia, poprzez przygotowanie do podania aż do podania pacjenta (np. system kodowania leków, który weryfikuje i zapamiętuje lek podany indywidualnemu pacjentowi) 1 (brak konieczności)	wprowadzenie systemu śledzącego lek w szpitalu od zlecenia, poprzez przygotowanie do podania aż do podania pacjenta (np. system kodowania leków, który weryfikuje i zapamiętuje lek podany indywidualnemu pacjentowi) 2	wprowadzenie systemu śledzącego lek w szpitalu od zlecenia, poprzez przygotowanie do podania aż do podania pacjenta (np. system kodowania leków, który weryfikuje i zapamiętuje lek podany indywidualnemu pacjentowi) 3	wprowadzenie systemu śledzącego lek w szpitalu od zlecenia, poprzez przygotowanie do podania aż do podania pacjenta (np. system kodowania leków, który weryfikuje i zapamiętuje lek podany indywidualnemu pacjentowi) 4	wprowadzenie systemu śledzącego lek w szpitalu od zlecenia, poprzez przygotowanie do podania aż do podania pacjenta (np. system kodowania leków, który weryfikuje i zapamiętuje lek podany indywidualnemu pacjentowi) 5 (bezwzględna konieczność)
cyfryzacja procesów w zakresie farmakoterapii w szpitalu (elektroniczne karty zleceń lekarskich, elektroniczne zapotrzebowania do apteki szpitalnej, elektroniczne zamówienia itp.)	cyfryzacja procesów w zakresie farmakoterapii w szpitalu (elektroniczne karty zleceń lekarskich, elektroniczne zapotrzebowania do apteki szpitalnej, elektroniczne zamówienia itp.) 1 (brak konieczności)	cyfryzacja procesów w zakresie farmakoterapii w szpitalu (elektroniczne karty zleceń lekarskich, elektroniczne zapotrzebowania do apteki szpitalnej, elektroniczne zamówienia itp.) 2	cyfryzacja procesów w zakresie farmakoterapii w szpitalu (elektroniczne karty zleceń lekarskich, elektroniczne zapotrzebowania do apteki szpitalnej, elektroniczne zamówienia itp.) 3	cyfryzacja procesów w zakresie farmakoterapii w szpitalu (elektroniczne karty zleceń lekarskich, elektroniczne zapotrzebowania do apteki szpitalnej, elektroniczne zamówienia itp.) 4	cyfryzacja procesów w zakresie farmakoterapii w szpitalu (elektroniczne karty zleceń lekarskich, elektroniczne zapotrzebowania do apteki szpitalnej, elektroniczne zamówienia itp.) 5 (bezwzględna konieczność)
programy edukacyjne w obszarze oceny ryzyka oraz zarządzania jakością dla farmaceutów szpitalnych (np. zarządzanie procesowe, rozwiązania lean management)	programy edukacyjne w obszarze oceny ryzyka oraz zarządzania jakością dla farmaceutów szpitalnych (np. zarządzanie procesowe, rozwiązania lean management) 1 (brak konieczności)	programy edukacyjne w obszarze oceny ryzyka oraz zarządzania jakością dla farmaceutów szpitalnych (np. zarządzanie procesowe, rozwiązania lean management) 2	programy edukacyjne w obszarze oceny ryzyka oraz zarządzania jakością dla farmaceutów szpitalnych (np. zarządzanie procesowe, rozwiązania lean management) 3	programy edukacyjne w obszarze oceny ryzyka oraz zarządzania jakością dla farmaceutów szpitalnych (np. zarządzanie procesowe, rozwiązania lean management) 4	programy edukacyjne w obszarze oceny ryzyka oraz zarządzania jakością dla farmaceutów szpitalnych (np. zarządzanie procesowe, rozwiązania lean management) 5

	1 (brak konieczności)	2	3	4	5 (bezwzględna konieczność)
					(bezwzględna konieczność)
opracowanie i wprowadzenie ogólnopolskich standardów jakościowych w farmacji szpitalnej, regulujących zasady postępowania w poszczególnych działach	opracowanie i wprowadzenie ogólnopolskich standardów jakościowych w farmacji szpitalnej, regulujących zasady postępowania w poszczególnych działach 1 (brak konieczności)	opracowanie i wprowadzenie ogólnopolskich standardów jakościowych w farmacji szpitalnej, regulujących zasady postępowania w poszczególnych działach 2	opracowanie i wprowadzenie ogólnopolskich standardów jakościowych w farmacji szpitalnej, regulujących zasady postępowania w poszczególnych działach 3	opracowanie i wprowadzenie ogólnopolskich standardów jakościowych w farmacji szpitalnej, regulujących zasady postępowania w poszczególnych działach 4	opracowanie i wprowadzenie ogólnopolskich standardów jakościowych w farmacji szpitalnej, regulujących zasady postępowania w poszczególnych działach 5 (bezwzględna konieczność)
opracowanie nowych wymogów dla pomieszczeń apteki szpitalnej i działu farmacji szpitalnej, biorących pod uwagę aktualny rozwój farmacji	opracowanie nowych wymogów dla pomieszczeń apteki szpitalnej i działu farmacji szpitalnej, biorących pod uwagę aktualny rozwój farmacji 1 (brak konieczności)	opracowanie nowych wymogów dla pomieszczeń apteki szpitalnej i działu farmacji szpitalnej, biorących pod uwagę aktualny rozwój farmacji 2	opracowanie nowych wymogów dla pomieszczeń apteki szpitalnej i działu farmacji szpitalnej, biorących pod uwagę aktualny rozwój farmacji 3	opracowanie nowych wymogów dla pomieszczeń apteki szpitalnej i działu farmacji szpitalnej, biorących pod uwagę aktualny rozwój farmacji 4	opracowanie nowych wymogów dla pomieszczeń apteki szpitalnej i działu farmacji szpitalnej, biorących pod uwagę aktualny rozwój farmacji 5 (bezwzględna konieczność)

14. Jeżeli uważa Pani/Pan, że potrzebne są inne zmiany w obszarze dystrybucyjnym, proszę o ich wpisanie: 0

* 15. Proszę o ocenę w skali 1-10 Pani/Pana **gotowości** do podjęcia pracy nad wprowadzeniem nowych rozwiązań w obszarze dystrybucyjnym, gdzie 1 oznacza brak gotowości a 10 pełną gotowość. 0

0 (brak gotowości) 5 10 (pełna gotowość)

NEW QUESTION

or Copy and paste questions

Wstecz Dalej

Obsługiwane przez
Zobacz, jak łatwo jest utworzyć ankietę.

0 z 37 z odpowiedziami

NEW PAGE

P4: Zmiana w obszarze sporządzania

-

Page Logic More Actions

UPGRADE TO ADD A LOGO

Zmiana w zawodzie farmaceuty szpitalnego

4. Zmiana w obszarze sporządzania

* 16. Czy uważa Pani/Pan, że konieczne jest wprowadzenie zmian w sposobie funkcjonowania farmaceutów w obszarze **sporządzania produktów leczniczych**? 0

- TAK
- NIE

* 17. Proszę o ocenę w skali 1-5 **konieczności** wprowadzenia następujących zmian w obszarze sporządzania produktów leczniczych, gdzie 1 oznacza brak konieczności a 5 bezwzględną konieczność wprowadzenia zmiany: 0

	1 (brak konieczności)	2	3	4	5 (bezwzględna konieczność)
wprowadzenie metodologii oceny ryzyka sporządzania jako narzędzia, które decyduje o postaci leku kupowanego przez szpital (RTA, RTU, lek wymagający przygotowania) oraz pomaga wskazać gdzie dany lek powinien zostać przygotowany do podania:	wprowadzenie metodologii oceny ryzyka sporządzania jako narzędzia, które decyduje o postaci leku kupowanego przez szpital (RTA, RTU, lek wymagający przygotowania) oraz pomaga wskazać gdzie dany lek powinien zostać przygotowany do podania: 1 (brak konieczności)	wprowadzenie metodologii oceny ryzyka sporządzania jako narzędzia, które decyduje o postaci leku kupowanego przez szpital (RTA, RTU, lek wymagający przygotowania) oraz pomaga wskazać gdzie dany lek powinien zostać przygotowany do podania: 2	wprowadzenie metodologii oceny ryzyka sporządzania jako narzędzia, które decyduje o postaci leku kupowanego przez szpital (RTA, RTU, lek wymagający przygotowania) oraz pomaga wskazać gdzie dany lek powinien zostać przygotowany do podania: 3	wprowadzenie metodologii oceny ryzyka sporządzania jako narzędzia, które decyduje o postaci leku kupowanego przez szpital (RTA, RTU, lek wymagający przygotowania) oraz pomaga wskazać gdzie dany lek powinien zostać przygotowany do podania: 4	wprowadzenie metodologii oceny ryzyka sporządzania jako narzędzia, które decyduje o postaci leku kupowanego przez szpital (RTA, RTU, lek wymagający przygotowania) oraz pomaga wskazać gdzie dany lek powinien zostać przygotowany do podania: 5 (bezwzględna konieczność)
wprowadzenie opłaty taxa laborum za sporządzanie jałowych produktów leczniczych w aptekach szpitalnych:	wprowadzenie opłaty taxa laborum za sporządzanie jałowych produktów leczniczych w aptekach szpitalnych: 1 (brak konieczności)	wprowadzenie opłaty taxa laborum za sporządzanie jałowych produktów leczniczych w aptekach szpitalnych: 2	wprowadzenie opłaty taxa laborum za sporządzanie jałowych produktów leczniczych w aptekach szpitalnych: 3	wprowadzenie opłaty taxa laborum za sporządzanie jałowych produktów leczniczych w aptekach szpitalnych: 4	wprowadzenie opłaty taxa laborum za sporządzanie jałowych produktów leczniczych w aptekach szpitalnych: 5 (bezwzględna konieczność)
umożliwienie rozliczania z NFZ nadmiarów leków finansowanych bezpośrednio (cytostatyki, leki w programach lekowych):	umożliwienie rozliczania z NFZ nadmiarów leków finansowanych bezpośrednio (cytostatyki, leki w programach lekowych): 1 (brak konieczności)	umożliwienie rozliczania z NFZ nadmiarów leków finansowanych bezpośrednio (cytostatyki, leki w programach lekowych): 2	umożliwienie rozliczania z NFZ nadmiarów leków finansowanych bezpośrednio (cytostatyki, leki w programach lekowych): 3	umożliwienie rozliczania z NFZ nadmiarów leków finansowanych bezpośrednio (cytostatyki, leki w programach lekowych): 4	umożliwienie rozliczania z NFZ nadmiarów leków finansowanych bezpośrednio (cytostatyki, leki w programach lekowych): 5 (bezwzględna konieczność)

18. Jeżeli uważa Pani/Pan, że potrzebne są inne zmiany w obszarze sporządzania, proszę o ich wpisanie: 0

* 19. Proszę o ocenę w skali 1-10 Pani/Pana **gotowości** do wprowadzenia nowych rozwiązań w obszarze sporządzania produktów leczniczych: [1-10] 0

0 (brak gotowości) 5 10 (pełna gotowość)

NEW QUESTION

or Copy and paste questions

Wstecz Dalej

Obsługiwane przez
Zobacz, jak łatwo jest utworzyć ankietę.

NEW PAGE

P5: Badania i rozwój

-

Page Logic More Actions

UPGRADE TO ADD A LOGO

Zmiana w zawodzie farmaceuty szpitalnego

5. Badania i rozwój

* 20. Czy uważa Pan/Pani, że w polskich aptekach szpitalnych **należy prowadzić prace badawcze i rozwojowe (B+R),** np. we współpracy z uniwersytetem lub firmą prywatną? Przykładowe obszary to technologia postaci leku, czy nowe technologie lekowe. 0

- TAK
- NIE

* 21. Proszę o ocenę w skali 1-5 **konieczności** wprowadzenia następujących zmian w obszarze badawczo-rozwojowym (B+R), gdzie 1 oznacza brak konieczności a 5 bezwzględną konieczność wprowadzenia zmiany: 0

	1 (brak konieczności)	2	3	4	5 (bezwzględna konieczność)
sporządzanie innowacyjnych produktów leczniczych w aptekach szpitalnych, np. produkty lecznicze terapii zaawansowanej (m.in. terapia genowa, terapia CAR-T):	sporządzanie innowacyjnych produktów leczniczych w aptekach szpitalnych, np. produkty lecznicze terapii zaawansowanej (m.in. terapia genowa, terapia CAR-T): 1 (brak konieczności)	sporządzanie innowacyjnych produktów leczniczych w aptekach szpitalnych, np. produkty lecznicze terapii zaawansowanej (m.in. terapia genowa, terapia CAR-T): 2	sporządzanie innowacyjnych produktów leczniczych w aptekach szpitalnych, np. produkty lecznicze terapii zaawansowanej (m.in. terapia genowa, terapia CAR-T): 3	sporządzanie innowacyjnych produktów leczniczych w aptekach szpitalnych, np. produkty lecznicze terapii zaawansowanej (m.in. terapia genowa, terapia CAR-T): 4	sporządzanie innowacyjnych produktów leczniczych w aptekach szpitalnych, np. produkty lecznicze terapii zaawansowanej (m.in. terapia genowa, terapia CAR-T): 5 (bezwzględna konieczność)
badania w obszarze innowacyjnych postaci leku i indywidualizacji dawki (np. druk 3D leków):	badania w obszarze innowacyjnych postaci leku i indywidualizacji dawki (np. druk 3D leków): 1 (brak konieczności)	badania w obszarze innowacyjnych postaci leku i indywidualizacji dawki (np. druk 3D leków): 2	badania w obszarze innowacyjnych postaci leku i indywidualizacji dawki (np. druk 3D leków): 3	badania w obszarze innowacyjnych postaci leku i indywidualizacji dawki (np. druk 3D leków): 4	badania w obszarze innowacyjnych postaci leku i indywidualizacji dawki (np. druk 3D leków): 5 (bezwzględna konieczność)
współpraca z uniwersytetami w zakresie B+R w obszarach dotyczących farmakoterapii:	współpraca z uniwersytetami w zakresie B+R w obszarach dotyczących farmakoterapii: 1 (brak konieczności)	współpraca z uniwersytetami w zakresie B+R w obszarach dotyczących farmakoterapii: 2	współpraca z uniwersytetami w zakresie B+R w obszarach dotyczących farmakoterapii: 3	współpraca z uniwersytetami w zakresie B+R w obszarach dotyczących farmakoterapii: 4	współpraca z uniwersytetami w zakresie B+R w obszarach dotyczących farmakoterapii: 5 (bezwzględna konieczność)

	1 (brak konieczności)	2	3	4	5 (bezwzględna konieczność)
współpraca z firmami prywatnymi w zakresie B+R w obszarach dotyczących farmakoterapii:	współpraca z firmami prywatnymi w zakresie B+R w obszarach dotyczących farmakoterapii: 1 (brak konieczności)	współpraca z firmami prywatnymi w zakresie B+R w obszarach dotyczących farmakoterapii: 2	współpraca z firmami prywatnymi w zakresie B+R w obszarach dotyczących farmakoterapii: 3	współpraca z firmami prywatnymi w zakresie B+R w obszarach dotyczących farmakoterapii: 4	współpraca z firmami prywatnymi w zakresie B+R w obszarach dotyczących farmakoterapii: 5 (bezwzględna konieczność)
udział w badaniach klinicznych produktów leczniczych	udział w badaniach klinicznych produktów leczniczych 1 (brak konieczności)	udział w badaniach klinicznych produktów leczniczych 2	udział w badaniach klinicznych produktów leczniczych 3	udział w badaniach klinicznych produktów leczniczych 4	udział w badaniach klinicznych produktów leczniczych 5 (bezwzględna konieczność)
udział w badaniach klinicznych wyrobów medycznych	udział w badaniach klinicznych wyrobów medycznych 1 (brak konieczności)	udział w badaniach klinicznych wyrobów medycznych 2	udział w badaniach klinicznych wyrobów medycznych 3	udział w badaniach klinicznych wyrobów medycznych 4	udział w badaniach klinicznych wyrobów medycznych 5 (bezwzględna konieczność)

22. Jeżeli uważa Pani/Pan, że potrzebne są inne zmiany w obszarze B+R, proszę o ich wpisanie: 0

* 23. Proszę o ocenę w skali 1-10 Pani/Pana **gotowości** do wprowadzenia nowych rozwiązań w obszarze badawczo-rozwojowym: 0

0 (brak gotowości) 5 10 (pełna gotowość)

NEW QUESTION

or Copy and paste questions

Wstecz Dalej

Obsługiwane przez
Zobacz, jak łatwo jest utworzyć ankietę.

0 z 37 z odpowiedziami

NEW PAGE

P6: Farmacja kliniczna - sekcja ogólna

-

Page Logic More Actions

UPGRADE TO ADD A LOGO

Zmiana w zawodzie farmaceuty szpitalnego

6. Farmacja kliniczna - sekcja ogólna

* 24. Czy uważa Pani/Pan, że konieczne jest wprowadzenie zmiany w sposobie funkcjonowania farmaceutów w szpitalu poprzez **uruchomienie usług farmacji klinicznej?** 0

- TAK
- NIE

* 25. Proszę o ocenę w skali 1-10 Pani/Pana ogólnej **gotowości** do podjęcia pracy nad uruchomieniem usług farmacji klinicznej w Państwa szpitalu: 0

0 (brak gotowości) 5 10 (pełna gotowość)

* 26. Czy w Pani/Pana ocenie posiada Pani/Pan **wiedzę** niezbędną do rozpoczęcia świadczenia usług farmacji klinicznej? 0

- TAK
- NIE

* 27. Czy w Pani/Pana ocenie obecnie posiada Pani/Pan **umiejętności** niezbędne do rozpoczęcia świadczenia usług farmacji klinicznej? 0

- TAK
- NIE

* 28. Czy w Pani/Pana ocenie obecnie posiada Pani/Pan **narzędzia (np. procedura, instrukcja, kwestionariusz dla pacjenta)** niezbędne do rozpoczęcia świadczenia usług farmacji klinicznej? 0

- TAK
- NIE

* 29. Czy w pracy posiada Pani/Pan **czas** niezbędny do rozpoczęcia i prowadzenia w Państwa miejscu pracy usług farmacji klinicznej? 0

- TAK
- NIE

* 30. Gdzie wg Pani/Pana powinien być zatrudniony farmaceuta kliniczny: 0

- w aptece szpitalnej (podlega kierownikowi apteki)
- w aptece szpitalnej pod warunkiem wydzielenia zespołu farmaceutów klinicznych i zapewnienia pracy wyłącznie w tym obszarze (podlega kierownikowi apteki)
- bezpośrednio w oddziale (podlega ordynatorowi oddziału)
- w odrębnym zespole farmacji klinicznej, który świadczy usługi dla całego szpitala (farmaceuta kliniczny podlega farmaceucie, który jest kierownikiem tego zespołu).

NEW QUESTION

or Copy and paste questions

Wstecz Dalej

Obsługiwane przez
Zobacz, jak łatwo jest utworzyć ankietę.

NEW PAGE

P7: Farmacja kliniczna - usługi

-

Page Logic More Actions

UPGRADE TO ADD A LOGO

Zmiana w zawodzie farmaceuty szpitalnego

7. Farmacja kliniczna - usługi

* 31. Proszę o ocenę Pani/Pana **znajomości** poszczególnych narzędzi farmacji klinicznej, gdzie 1 oznacza brak znajomości a 5 pełną znajomość narzędzia: 0

	1 (brak znajomości)	2	3	4	5 (pełna znajomość)
Koncyliacja lekowa	Koncyliacja lekowa 1 (brak znajomości)	Koncyliacja lekowa 2	Koncyliacja lekowa 3	Koncyliacja lekowa 4	Koncyliacja lekowa 5 (pełna znajomość)
Przegląd lekowy	Przegląd lekowy 1 (brak znajomości)	Przegląd lekowy 2	Przegląd lekowy 3	Przegląd lekowy 4	Przegląd lekowy 5 (pełna znajomość)
Opracowanie indywidualnego planu opieki farmaceutycznej	Opracowanie indywidualnego planu opieki farmaceutycznej 1 (brak znajomości)	Opracowanie indywidualnego planu opieki farmaceutycznej 2	Opracowanie indywidualnego planu opieki farmaceutycznej 3	Opracowanie indywidualnego planu opieki farmaceutycznej 4	Opracowanie indywidualnego planu opieki farmaceutycznej 5 (pełna znajomość)
Edukacja w obszarze farmakoterapii kierowana do pacjentów lub personelu medycznego	Edukacja w obszarze farmakoterapii kierowana do pacjentów lub personelu medycznego 1 (brak znajomości)	Edukacja w obszarze farmakoterapii kierowana do pacjentów lub personelu medycznego 2	Edukacja w obszarze farmakoterapii kierowana do pacjentów lub personelu medycznego 3	Edukacja w obszarze farmakoterapii kierowana do pacjentów lub personelu medycznego 4	Edukacja w obszarze farmakoterapii kierowana do pacjentów lub personelu medycznego 5 (pełna znajomość)
Prowadzenie terapii monitorowanej stężeniem leku we krwi	Prowadzenie terapii monitorowanej stężeniem leku we krwi 1 (brak znajomości)	Prowadzenie terapii monitorowanej stężeniem leku we krwi 2	Prowadzenie terapii monitorowanej stężeniem leku we krwi 3	Prowadzenie terapii monitorowanej stężeniem leku we krwi 4	Prowadzenie terapii monitorowanej stężeniem leku we krwi 5 (pełna znajomość)
Indywidualna konsultacja dotycząca farmakoterapii pacjenta na zlecenie lekarza	Indywidualna konsultacja dotycząca farmakoterapii pacjenta na zlecenie lekarza 1 (brak znajomości)	Indywidualna konsultacja dotycząca farmakoterapii pacjenta na zlecenie lekarza 2	Indywidualna konsultacja dotycząca farmakoterapii pacjenta na zlecenie lekarza 3	Indywidualna konsultacja dotycząca farmakoterapii pacjenta na zlecenie lekarza 4	Indywidualna konsultacja dotycząca farmakoterapii pacjenta na zlecenie lekarza 5 (pełna znajomość)
Kreowanie szpitalnej polityki antybiotykowej	Kreowanie szpitalnej polityki antybiotykowej 1 (brak znajomości)	Kreowanie szpitalnej polityki antybiotykowej 2	Kreowanie szpitalnej polityki antybiotykowej 3	Kreowanie szpitalnej polityki antybiotykowej 4	Kreowanie szpitalnej polityki antybiotykowej 5 (pełna znajomość)
Konsultowanie indywidualnej antybiotykoterapii pacjenta	Konsultowanie indywidualnej antybiotykoterapii pacjenta 1 (brak znajomości)	Konsultowanie indywidualnej antybiotykoterapii pacjenta 2	Konsultowanie indywidualnej antybiotykoterapii pacjenta 3	Konsultowanie indywidualnej antybiotykoterapii pacjenta 4	Konsultowanie indywidualnej antybiotykoterapii pacjenta 5 (pełna znajomość)
Uczestnictwo w obchodach na oddziałach i konsyliach	Uczestnictwo w obchodach na oddziałach i	Uczestnictwo w obchodach na oddziałach i	Uczestnictwo w obchodach na oddziałach i	Uczestnictwo w obchodach na oddziałach i	Uczestnictwo w obchodach na oddziałach i

	1 (brak znajomości)	2	3	4	5 (pełna znajomość)
interdyscyplinarnych	konsyliach interdyscyplinarnych 1 (brak znajomości)	konsyliach interdyscyplinarnych 2	konsyliach interdyscyplinarnych 3	konsyliach interdyscyplinarnych 4	konsyliach interdyscyplinarnych 5 (pełna znajomość)
Wystawianie recept kontynuacyjnych, np. dotyczących podania kolejnego cyklu chemioterapii pacjentowi (zgodnie ze schematem zleconym przez lekarza):	Wystawianie recept kontynuacyjnych, np. dotyczących podania kolejnego cyklu chemioterapii pacjentowi (zgodnie ze schematem zleconym przez lekarza): 1 (brak znajomości)	Wystawianie recept kontynuacyjnych, np. dotyczących podania kolejnego cyklu chemioterapii pacjentowi (zgodnie ze schematem zleconym przez lekarza): 2	Wystawianie recept kontynuacyjnych, np. dotyczących podania kolejnego cyklu chemioterapii pacjentowi (zgodnie ze schematem zleconym przez lekarza): 3	Wystawianie recept kontynuacyjnych, np. dotyczących podania kolejnego cyklu chemioterapii pacjentowi (zgodnie ze schematem zleconym przez lekarza): 4	Wystawianie recept kontynuacyjnych, np. dotyczących podania kolejnego cyklu chemioterapii pacjentowi (zgodnie ze schematem zleconym przez lekarza): 5 (pełna znajomość)

* 32. Proszę o ocenę w skali 1-5 **konieczności** wprowadzenia następujących usług farmacji klinicznej, gdzie 1 oznacza brak konieczności a 5 bezwzględną konieczność wprowadzenia narzędzia: 0

	1 (brak konieczności)	2	3	4	5 (bezwzględna konieczność)
koncyliacja lekowa	koncyliacja lekowa 1 (brak konieczności)	koncyliacja lekowa 2	koncyliacja lekowa 3	koncyliacja lekowa 4	koncyliacja lekowa 5 (bezwzględna konieczność)
przegląd lekowy	przegląd lekowy 1 (brak konieczności)	przegląd lekowy 2	przegląd lekowy 3	przegląd lekowy 4	przegląd lekowy 5 (bezwzględna konieczność)
opracowanie indywidualnego planu opieki farmaceutycznej	opracowanie indywidualnego planu opieki farmaceutycznej 1 (brak konieczności)	opracowanie indywidualnego planu opieki farmaceutycznej 2	opracowanie indywidualnego planu opieki farmaceutycznej 3	opracowanie indywidualnego planu opieki farmaceutycznej 4	opracowanie indywidualnego planu opieki farmaceutycznej 5 (bezwzględna konieczność)
edukacja w obszarze farmakoterapii kierowana do pacjentów lub personelu medycznego	edukacja w obszarze farmakoterapii kierowana do pacjentów lub personelu medycznego 1 (brak konieczności)	edukacja w obszarze farmakoterapii kierowana do pacjentów lub personelu medycznego 2	edukacja w obszarze farmakoterapii kierowana do pacjentów lub personelu medycznego 3	edukacja w obszarze farmakoterapii kierowana do pacjentów lub personelu medycznego 4	edukacja w obszarze farmakoterapii kierowana do pacjentów lub personelu medycznego 5 (bezwzględna konieczność)
prowadzenie terapii monitorowanej stężeniem leku we krwi	prowadzenie terapii monitorowanej stężeniem leku we krwi 1 (brak konieczności)	prowadzenie terapii monitorowanej stężeniem leku we krwi 2	prowadzenie terapii monitorowanej stężeniem leku we krwi 3	prowadzenie terapii monitorowanej stężeniem leku we krwi 4	prowadzenie terapii monitorowanej stężeniem leku we krwi 5 (bezwzględna konieczność)
indywidualna konsultacja dotycząca farmakoterapii pacjenta na zlecenie lekarza	indywidualna konsultacja dotycząca farmakoterapii pacjenta na zlecenie lekarza 1 (brak konieczności)	indywidualna konsultacja dotycząca farmakoterapii pacjenta na zlecenie lekarza 2	indywidualna konsultacja dotycząca farmakoterapii pacjenta na zlecenie lekarza 3	indywidualna konsultacja dotycząca farmakoterapii pacjenta na zlecenie lekarza 4	indywidualna konsultacja dotycząca farmakoterapii pacjenta na zlecenie lekarza 5 (bezwzględna konieczność)
kreowanie szpitalnej polityki antybiotykowej	kreowanie szpitalnej polityki	kreowanie szpitalnej polityki antybiotykowej 2	kreowanie szpitalnej polityki antybiotykowej 3	kreowanie szpitalnej polityki antybiotykowej 4	kreowanie szpitalnej polityki antybiotykowej 5

	1 (brak konieczności)	2	3	4	5 (bezwzględna konieczność)
	antybiotykowej 1 (brak konieczności)				(bezwzględna konieczność)
konsultowanie indywidualnej antybiotykoterapii pacjenta	konsultowanie indywidualnej antybiotykoterapii pacjenta 1 (brak konieczności)	konsultowanie indywidualnej antybiotykoterapii pacjenta 2	konsultowanie indywidualnej antybiotykoterapii pacjenta 3	konsultowanie indywidualnej antybiotykoterapii pacjenta 4	konsultowanie indywidualnej antybiotykoterapii pacjenta 5 (bezwzględna konieczność)
uczestnictwo w obchodach na oddziałach i konsyliach interdyscyplinarnych	uczestnictwo w obchodach na oddziałach i konsyliach interdyscyplinarnych 1 (brak konieczności)	uczestnictwo w obchodach na oddziałach i konsyliach interdyscyplinarnych 2	uczestnictwo w obchodach na oddziałach i konsyliach interdyscyplinarnych 3	uczestnictwo w obchodach na oddziałach i konsyliach interdyscyplinarnych 4	uczestnictwo w obchodach na oddziałach i konsyliach interdyscyplinarnych 5 (bezwzględna konieczność)
wystawianie recept kontynuacyjnych, np. dotyczących podania kolejnego cyklu chemioterapii pacjentowi (zgodnie ze schematem zleconym przez lekarza):	wystawianie recept kontynuacyjnych, np. dotyczących podania kolejnego cyklu chemioterapii pacjentowi (zgodnie ze schematem zleconym przez lekarza): 1 (brak konieczności)	wystawianie recept kontynuacyjnych, np. dotyczących podania kolejnego cyklu chemioterapii pacjentowi (zgodnie ze schematem zleconym przez lekarza): 2	wystawianie recept kontynuacyjnych, np. dotyczących podania kolejnego cyklu chemioterapii pacjentowi (zgodnie ze schematem zleconym przez lekarza): 3	wystawianie recept kontynuacyjnych, np. dotyczących podania kolejnego cyklu chemioterapii pacjentowi (zgodnie ze schematem zleconym przez lekarza): 4	wystawianie recept kontynuacyjnych, np. dotyczących podania kolejnego cyklu chemioterapii pacjentowi (zgodnie ze schematem zleconym przez lekarza): 5 (bezwzględna konieczność)

33. Proszę o ocenę w skali 1-5 Pani/Pana **gotowości** do wprowadzenia następujących usług farmacji klinicznej w Pani/Pana szpitalu, , gdzie 1 oznacza brak gotowości a 5 pełną gotowość do wprowadzenia zmiany: 0

	1 (brak gotowości)	2	3	4	5 (pełna gotowość)
koncyliacja lekowa	koncyliacja lekowa 1 (brak gotowości)	koncyliacja lekowa 2	koncyliacja lekowa 3	koncyliacja lekowa 4	koncyliacja lekowa 5 (pełna gotowość)
przegląd lekowy	przegląd lekowy 1 (brak gotowości)	przegląd lekowy 2	przegląd lekowy 3	przegląd lekowy 4	przegląd lekowy 5 (pełna gotowość)
opracowanie indywidualnego planu opieki farmaceutycznej	opracowanie indywidualnego planu opieki farmaceutycznej 1 (brak gotowości)	opracowanie indywidualnego planu opieki farmaceutycznej 2	opracowanie indywidualnego planu opieki farmaceutycznej 3	opracowanie indywidualnego planu opieki farmaceutycznej 4	opracowanie indywidualnego planu opieki farmaceutycznej 5 (pełna gotowość)
edukacja w obszarze farmakoterapii kierowana do pacjentów lub personelu medycznego	edukacja w obszarze farmakoterapii kierowana do pacjentów lub personelu medycznego 1 (brak gotowości)	edukacja w obszarze farmakoterapii kierowana do pacjentów lub personelu medycznego 2	edukacja w obszarze farmakoterapii kierowana do pacjentów lub personelu medycznego 3	edukacja w obszarze farmakoterapii kierowana do pacjentów lub personelu medycznego 4	edukacja w obszarze farmakoterapii kierowana do pacjentów lub personelu medycznego 5 (pełna gotowość)
prowadzenie terapii monitorowanej stężeniem leku we krwi	prowadzenie terapii monitorowanej stężeniem leku we krwi 1 (brak gotowości)	prowadzenie terapii monitorowanej stężeniem leku we krwi 2	prowadzenie terapii monitorowanej stężeniem leku we krwi 3	prowadzenie terapii monitorowanej stężeniem leku we krwi 4	prowadzenie terapii monitorowanej stężeniem leku we krwi 5 (pełna gotowość)
indywidualna konsultacja dotycząca farmakoterapii	indywidualna konsultacja dotycząca farmakoterapii	indywidualna konsultacja dotycząca farmakoterapii	indywidualna konsultacja dotycząca farmakoterapii	indywidualna konsultacja dotycząca farmakoterapii	indywidualna konsultacja dotycząca farmakoterapii

	1 (brak gotowości)	2	3	4	5 (pełna gotowość)
pacjenta na zlecenie lekarza	pacjenta na zlecenie lekarza 1 (brak gotowości)	pacjenta na zlecenie lekarza 2	pacjenta na zlecenie lekarza 3	pacjenta na zlecenie lekarza 4	pacjenta na zlecenie lekarza 5 (pełna gotowość)
kreowanie szpitalnej polityki antybiotykowej	kreowanie szpitalnej polityki antybiotykowej 1 (brak gotowości)	kreowanie szpitalnej polityki antybiotykowej 2	kreowanie szpitalnej polityki antybiotykowej 3	kreowanie szpitalnej polityki antybiotykowej 4	kreowanie szpitalnej polityki antybiotykowej 5 (pełna gotowość)
konsultowanie indywidualnej antybiotykoterapii pacjenta	konsultowanie indywidualnej antybiotykoterapii pacjenta 1 (brak gotowości)	konsultowanie indywidualnej antybiotykoterapii pacjenta 2	konsultowanie indywidualnej antybiotykoterapii pacjenta 3	konsultowanie indywidualnej antybiotykoterapii pacjenta 4	konsultowanie indywidualnej antybiotykoterapii pacjenta 5 (pełna gotowość)
uczestnictwo w obchodach na oddziałach i konsyliach interdyscyplinarnych	uczestnictwo w obchodach na oddziałach i konsyliach interdyscyplinarnych 1 (brak gotowości)	uczestnictwo w obchodach na oddziałach i konsyliach interdyscyplinarnych 2	uczestnictwo w obchodach na oddziałach i konsyliach interdyscyplinarnych 3	uczestnictwo w obchodach na oddziałach i konsyliach interdyscyplinarnych 4	uczestnictwo w obchodach na oddziałach i konsyliach interdyscyplinarnych 5 (pełna gotowość)
wystawianie recept kontynuacyjnych, np. dotyczących podania kolejnego cyklu chemioterapii pacjentowi (zgodnie ze schematem zleconym przez lekarza):	wystawianie recept kontynuacyjnych, np. dotyczących podania kolejnego cyklu chemioterapii pacjentowi (zgodnie ze schematem zleconym przez lekarza): 1 (brak gotowości)	wystawianie recept kontynuacyjnych, np. dotyczących podania kolejnego cyklu chemioterapii pacjentowi (zgodnie ze schematem zleconym przez lekarza): 2	wystawianie recept kontynuacyjnych, np. dotyczących podania kolejnego cyklu chemioterapii pacjentowi (zgodnie ze schematem zleconym przez lekarza): 3	wystawianie recept kontynuacyjnych, np. dotyczących podania kolejnego cyklu chemioterapii pacjentowi (zgodnie ze schematem zleconym przez lekarza): 4	wystawianie recept kontynuacyjnych, np. dotyczących podania kolejnego cyklu chemioterapii pacjentowi (zgodnie ze schematem zleconym przez lekarza): 5 (pełna gotowość)

34. Jeżeli uważa Pani/Pan, że warto wprowadzić również inne usługi farmacji klinicznej, proszę o podanie ich nazw: 0

NEW QUESTION

or Copy and paste questions

Wstecz Dalej

Obsługiwane przez
Zobacz, jak łatwo jest utworzyć ankietę.

0 z 37 z odpowiedziami

NEW PAGE

P8: Farmacja kliniczna - zakończenie

Page Logic More Actions

UPGRADE TO ADD A LOGO

Zmiana w zawodzie farmaceuty szpitalnego

8. Farmacja kliniczna - zakończenie

* 35. Czy obawia się Pani/Pan większej ilości pracy w związku z uruchomieniem nowych aktywności klinicznych farmaceutów? 0

TAK

NIE

0 UPGRADE TO EDIT OPTIONS LOGIC MOVE COPY LIBRARY DELETE

* 36. Proszę ocenić ewentualne **przeszkody** we wprowadzeniu usług farmacji klinicznej w polskich szpitalach, gdzie 1 oznacza, że nie jest to przeszkodą a 5 że jest to bariera, bez której pokonania nie będzie możliwa realizacja zmiany: 0

	1 (nie jest to przeszkodą)	2	3	4	5 (jest to przeszkoda blokująca zmianę)
brak wystarczającej ilości personelu fachowego	brak wystarczającej ilości personelu fachowego 1 (nie jest to przeszkodą)	brak wystarczającej ilości personelu fachowego 2	brak wystarczającej ilości personelu fachowego 3	brak wystarczającej ilości personelu fachowego 4	brak wystarczającej ilości personelu fachowego 5 (jest to przeszkoda blokująca zmianę)
brak wiedzy do świadczenia tego rodzaju usług przez farmaceutów	brak wiedzy do świadczenia tego rodzaju usług przez farmaceutów 1 (nie jest to przeszkodą)	brak wiedzy do świadczenia tego rodzaju usług przez farmaceutów 2	brak wiedzy do świadczenia tego rodzaju usług przez farmaceutów 3	brak wiedzy do świadczenia tego rodzaju usług przez farmaceutów 4	brak wiedzy do świadczenia tego rodzaju usług przez farmaceutów 5 (jest to przeszkoda blokująca zmianę)
brak umiejętności świadczenia tego rodzaju usług przez farmaceutów	brak umiejętności świadczenia tego rodzaju usług przez farmaceutów 1 (nie jest to przeszkodą)	brak umiejętności świadczenia tego rodzaju usług przez farmaceutów 2	brak umiejętności świadczenia tego rodzaju usług przez farmaceutów 3	brak umiejętności świadczenia tego rodzaju usług przez farmaceutów 4	brak umiejętności świadczenia tego rodzaju usług przez farmaceutów 5 (jest to przeszkoda blokująca zmianę)
brak finansowania usług farmacji klinicznej przez płatnika	brak finansowania usług farmacji klinicznej przez płatnika 1 (nie jest to przeszkodą)	brak finansowania usług farmacji klinicznej przez płatnika 2	brak finansowania usług farmacji klinicznej przez płatnika 3	brak finansowania usług farmacji klinicznej przez płatnika 4	brak finansowania usług farmacji klinicznej przez płatnika 5 (jest to przeszkoda blokująca zmianę)

37. Proszę o podanie innych ewentualnych przeszkód we wprowadzeniu usług farmacji klinicznej: 0

NEW QUESTION
